# Effect of very low-protein diets supplemented with branched-chain amino acids on energy balance, plasma metabolomics and fecal microbiome of pigs

**DOI:** 10.1038/s41598-020-72816-8

**Published:** 2020-09-28

**Authors:** Shelby Spring, Hasitha Premathilake, Chloe Bradway, Cedrick Shili, Udaya DeSilva, Scott Carter, Adel Pezeshki

**Affiliations:** Department of Animal and Food Sciences, Oklahoma State University, 206C Animal Science Building, Stillwater, OK 74078 USA

**Keywords:** Biochemistry, Physiology

## Abstract

Feeding pigs with very-low protein (VLP) diets while supplemented with limiting amino acids (AA) results in decreased growth. The objective of this study was to determine if supplementing VLP diets with branched-chain AA (BCAA) would reverse the negative effects of these diets on growth and whether this is associated with alterations in energy balance, blood metabolomics and fecal microbiota composition. Twenty-four nursery pigs were weight-matched, individually housed and allotted into following treatments (n = 8/group): control (CON), low protein (LP) and LP supplemented with BCAA (LP + BCAA) for 4 weeks. Relative to CON, pigs fed with LP had lower feed intake (FI) and body weight (BW) throughout the study, but those fed with LP + BCAA improved overall FI computed for 4 weeks, tended to increase the overall average daily gain, delayed the FI and BW depression for ~ 2 weeks and had transiently higher energy expenditure. Feeding pigs with LP + BCAA impacted the phenylalanine and protein metabolism and fatty acids synthesis pathways. Compared to CON, the LP + BCAA group had higher abundance of *Paludibacteraceae* and *Synergistaceae* and reduced populations of *Streptococcaceae, Oxyphotobacteria_unclassified, Pseudomonadaceae and Shewanellaceae* in their feces. Thus, supplementing VLP diets with BCAA temporarily annuls the adverse effects of these diets on growth, which is linked with alterations in energy balance and metabolic and gut microbiome profile.

## Introduction

One of the most stressful events in pigs’ life cycle is weaning piglets from the sow^1^. During weaning, piglets are exposed to multiple stressors such as the stresses associated with transportation and handling, transition from receiving highly palatable milk to less palatable and a complex dry feed, social hierarchy, new environment and etc. These together with developing multiple physiological and anatomical adaptions in digestive tract can lead to digestive dysfunctions and impaired immune system, which ultimately results in reduced growth and health in weaned nursery pigs^1^. Reducing the dietary protein content along with supplementation of limiting amino acids (AA) has been suggested to improve the animal’s health by reducing the digestive problems and incidence of diarrhea^2,3^. Further, due to the cost of dietary protein and environmental concerns, low protein diets have been proposed to be used in the swine industry to reduce the feed cost and nitrogen excretion^4–7^.

Decreasing the dietary protein level by less than 25% of recommendations of Nutrient Requirements of Swine^8^ and supplementing first four limiting AA, *i.e.* lysine, methionine, threonine and tryptophan while improving health do not appear to have negative effects on growth performance of young and growing-finishing pigs^6,9–13^. Moderate to severe reduction in dietary protein (> 25% reduction) may produce even more beneficial results in terms of health and nutrients excretion; however, these diets although supplemented with first limiting AA decrease feed efficiency in nursery and growing pigs^3,14–16^. Thus, there is a need to assess the effect of next limiting AA that can possibly prevent the reduction in growth performance of pigs fed with very low protein (VLP) diets.

Branched-chain AA (BCAA) including leucine, isoleucine and valine are essential AA. Isoleucine and valine are the next limiting AA after lysine, methionine, threonine and tryptophan for corn-soybean based diets^17–19^ and leucine is important for skeletal muscle protein synthesis in young pigs^20,21^. Supplementation of slightly low protein diets (17% crude protein [CP]) with all BCAA improved the growth performance of nursery^22–26^ and growing^27^ pigs. Dietary deficiency of valine decreased the average daily feed intake (ADFI) and growth performance of weaned pigs^17^ and supplementing slightly protein deprived diets with isoleucine and valine protected nursery and growing pigs against reduction in feed intake (FI) and body weight (BW)^7,19,28–30^. Further, the average daily gain (ADG) was increased with increasing the standard ileal digestibility (SID) valine:lysine in standard protein diets of nursery^31^ and growing pigs^32^. Thus, previous research provides evidence on beneficial effects of supplemental BCAA on growth of pigs fed with diets with normal or slightly low protein content.

We previously demonstrated that VLP diets (12% CP) decreased the serum isoleucine, valine and 2-ketoisocaproic acid, an intermediate in leucine catabolism and also greatly influenced degradation and endogenous biosynthesis pathways of BCAA^33^. In one study, growth performance of nursery pigs was improved when VLP diets (12 and 14% CP) supplemented with mixture of BCAA, histidine and phenylalanine^34^; however, due to using a combination of different AA, this improvement cannot be specifically attributed to BCAA. Thus, little is known whether BCAA can improve the performance of pigs when supplemented to VLP diets.

Previous research has shown that BCAA improve the growth performance of pigs fed with slightly low protein diets likely through improvement in metabolic profile in liver and muscle^28,35^, oxidative capacity of muscle^36^, plasma concentration of BCAA^22^, intestinal morphology and cell proliferation^37^, and protein metabolism and muscle growth^21,27,38^. Little is known about the effect of VLP diets supplemented with BCAA on energy balance as well as the mechanisms by which these diets influence the growth performance of nursery pigs. In particular, to the best of our knowledge no research has been conducted to assess the energy expenditure (EE), metabolomics profile and gut microbiota of pigs fed with VLP diets supplemented with BCAA. Therefore, the objective of this study was to determine the effect of VLP diets supplemented with BCAA on growth efficiency, energy balance, blood cytokines and metabolomics profile and fecal microbiota composition of nursery pigs.

## Results

### Feed intake, energy expenditure, respiratory quotient and body weight

**Figure 1:**
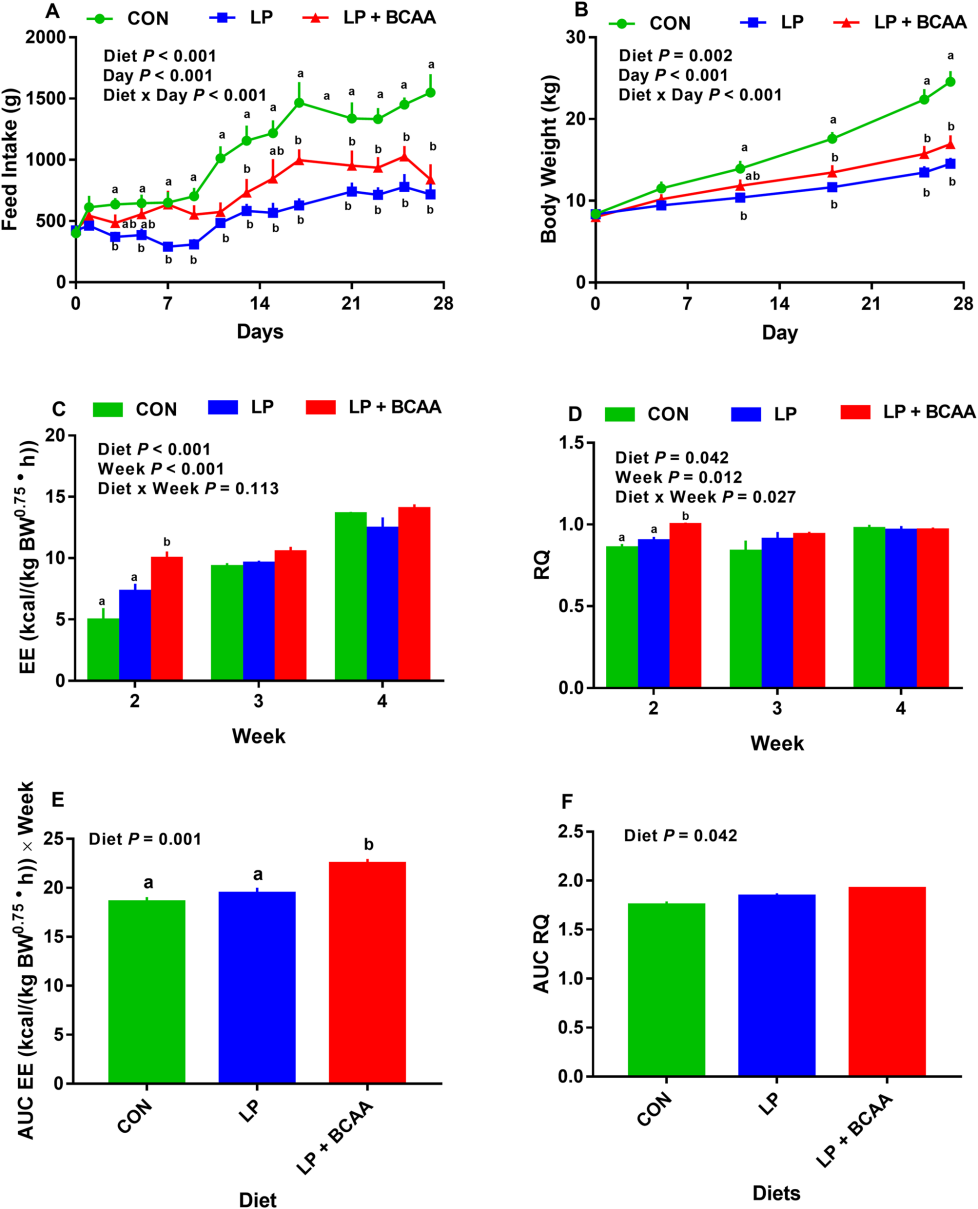
Effect of very low protein diets supplemented with branched-chain amino acids on energy balance. (**A**) feed intake, (**B**) body weight, (**C**) mean energy expenditure (EE), (**D**) mean respiratory quotient (RQ), (**E**) area under curve (AUC) for EE, and (**F**) AUC for RQ. CON, control diet; LP, low protein diet; LP + BCAA, low protein diet supplemented with branched-chain amino acids. Among groups, values with different superscripts are different (*P* ≤ 0.05). The values are means ± standard errors of means, n = 8.

**Figure 2:**
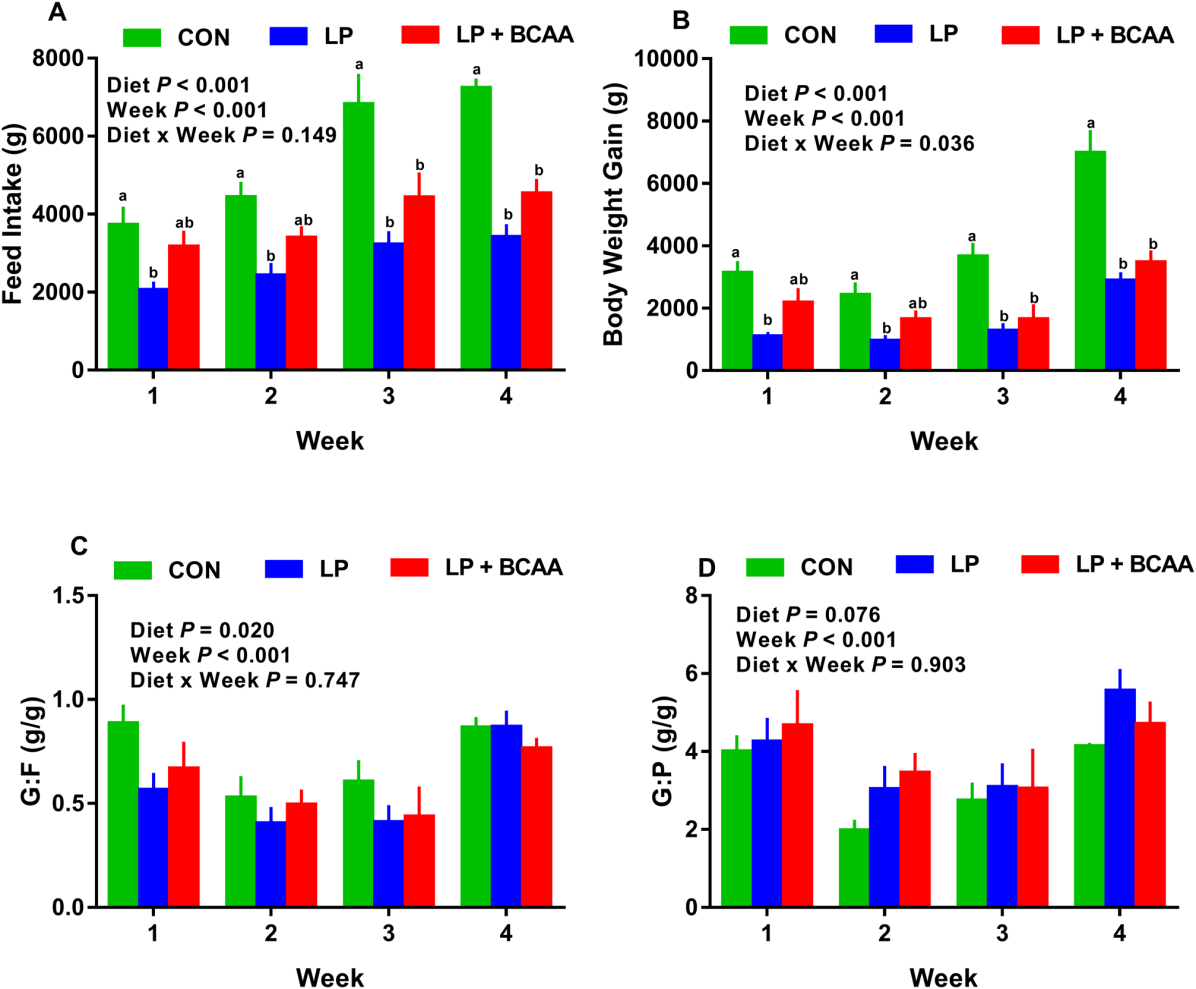
Effect of very low protein diets supplemented with branched-chain amino acids on weekly feed intake, body weight gain and feed and protein efficiency ratio. (**A**) cumulative feed intake, (**B**) body weight gain, (**C**) gain to feed ratio (G:F), and (**D**) gain to protein ratio (G:P). CON, control diet; LP, low protein diet; LP + BCAA, low protein diet supplemented with branched-chain amino acids. Among groups, values with different superscripts are different (*P* ≤ 0.05). The values are means ± standard errors of means, n = 8.

Overall, the effects of diet, day and the interaction of diet and day on FI were significant (*P* < 0.001; Fig. 1A). Compared to control (CON), low protein (LP) diet decreased FI by day (d) 3 onwards while pigs fed low protein diets supplemented with BCAA (LP + BCAA) did not reduce the FI until d 11 of the study (Fig. 1A). Relative to CON, LP + BCAA decreased FI after d 11 (*P* < 0.028; Fig. 1A). Except transient differences between LP and LP + BCAA for FI, no difference in FI was found between these two groups. Compared to CON, LP and LP + BCAA had lower overall ADFI by 50.6% and 31.9%, respectively during whole 4 weeks of study (Supplementary Table. S1). LP + BCAA had higher overall ADFI (*i.e.* during entire 4 weeks of study) by 37.9% compared to LP (Supplementary Table. S1). Overall, the effect of diet and week on weekly FI was significant (*P* < 0.001; Fig. 2A). However, the interaction of diet and week for weekly FI was not significant (*P* = 0.149; Fig. 2A). The LP had significantly lower weekly FI throughout the study when compared to CON (Fig. 2A). Interestingly, there was no significant difference between CON and LP + BCAA for FI during week (wk) 1 and 2 of the study (Fig. 2A), but LP + BCAA had significantly lower FI than CON on wk 3 and 4 (Fig. 2A). No significant differences in weekly FI were detected between LP and LP + BCAA throughout the study (Fig. 2A). Compared to CON, both LP and LP + BCAA had lower overall ADPI throughout the study (*P* < 0.001; Supplementary Table. S1). The LP + BCAA tended to increase the ADPI compared to LP (P = 0.054; Supplementary Table. S1).

Overall, the effect of diet and week on weekly EE was significant (*P* < 0.001; Fig. 1C). However, the interaction of diet and week on weekly EE was not significant (*P* = 0.113; Fig. 1C). Due to some technical problems with our calorimetery system, the EE data were not collected during first week of the study. Pigs fed LP + BCAA had higher EE during wk 2 relative to CON and LP (*P* = 0.001 and 0.017, respectively). After 2 wk, there was no significant difference in EE among all dietary treatments (Fig. 1C). When EE data were expressed as area under the curve (AUC), LP + BCAA had a higher AUC for EE compared to both CON and LP (*P* < 0.002; Fig. 1E). LP showed no difference in AUC for EE compared to CON (Fig. 1C,E). Overall, the effect of diet, week and the interaction of diet and week on weekly RQ was significant (*P* = 0.042, 0.012 and 0.027, respectively; Fig. 1D). LP + BCAA had significantly higher RQ during wk 2, compared to both CON and LP (*P* = 0.001 and 0.005, respectively; Fig. 2D). There were no differences in weekly RQ across experimental groups on wk 3 and 4 (Fig. 1D). Although the effect of diet on AUC for RQ was significant (*P* = 0.042), no significant differences in AUC for RQ were found among dietary treatments (Fig. 1F).

The initial BW did not differ across dietary treatments, with an average BW of 8.26 ± 0.34 kg (*P* = 0.832; Supplementary Table. S1). Compared to CON, both LP and LP + BCAA depressed the final BW by 40.8% and 31.0%, respectively (*P* < 0.0001; Supplementary Table. S1). No significant difference in final BW was detected between LP and LP + BCAA groups (*P* = 0.282; Supplementary Table. S1). Compared to CON, both LP and LP + BCAA had 61.8% and 44.9% lower overall ADG, respectively (Supplementary Table. S1). Interestingly, LP + BCAA tended to increase the overall ADG by 44.1% compared to LP (*P* = 0.076; Supplementary Table. S1). Overall, the effects of diet, day and the interaction of diet and day on BW were significant (*P* = 0.002, < 0.001, < 0.001 respectively; Fig. 1B). Relative to CON, LP reduced BW by d 11 onwards (*P* < 0.011; Fig. 1B). Pigs fed LP + BCAA did not reduce the BW until d 18 of the study compared to CON (*P* < 0.004; Fig. 1B). Pigs fed LP gained significantly less weight than CON throughout the study (Fig. 2B). Pigs received LP + BCAA did not reduce the BW gain in first two weeks of the study, but they gained less than CON on wk 3 and 4 (Fig. 2B). There was no significant difference between LP and LP + BCAA for weekly BW gain (Fig. 2B).

Overall G:F and G:P (*i.e.* for whole 4 weeks of study) were different among dietary treatments (*P* = 0.005 and 0.021, respectively; Supplementary Table. S1). Pigs fed LP and LP + BCAA reduced the overall G:F compared to CON (Supplementary Table. S1), but weekly G:F did not change among groups (Fig. 2C). The overall G:P for LP was significantly higher compared to CON (*P* = 0.024; Supplementary Table. S1), but that was not different compared to LP + BCAA. The overall G:P for LP + BCAA tended to be increased significantly relative to CON (*P* = 0.071; Supplementary Table. S1). The weekly G:P did not appear to change among dietary groups, but overall the effect of diet on G:P tended to be significant (*P* = 0.076; Fig. 2D).

### Plasma cytokines

The effect of diet on interleukin 12p40 (IL-12p40) concentration was significant (*P* = 0.038; Supplementary Fig. S1A). Pigs fed LP had a higher plasma concentration of IL-12p40 concentration than CON (Supplementary Fig. S1A). The plasma concentration of IL-12p40 for LP + BCAA did not differ from CON and LP (Supplementary Fig. S1A). The concentration of tumor necrosis factor-α (TNF-α) and interleukin-6 (IL-6) was not significantly different among dietary treatments (*P* = 0.312 and 0.138, respectively; Supplementary Fig. S1B and C).

### Plasma metabolomics

**Figure 3:**
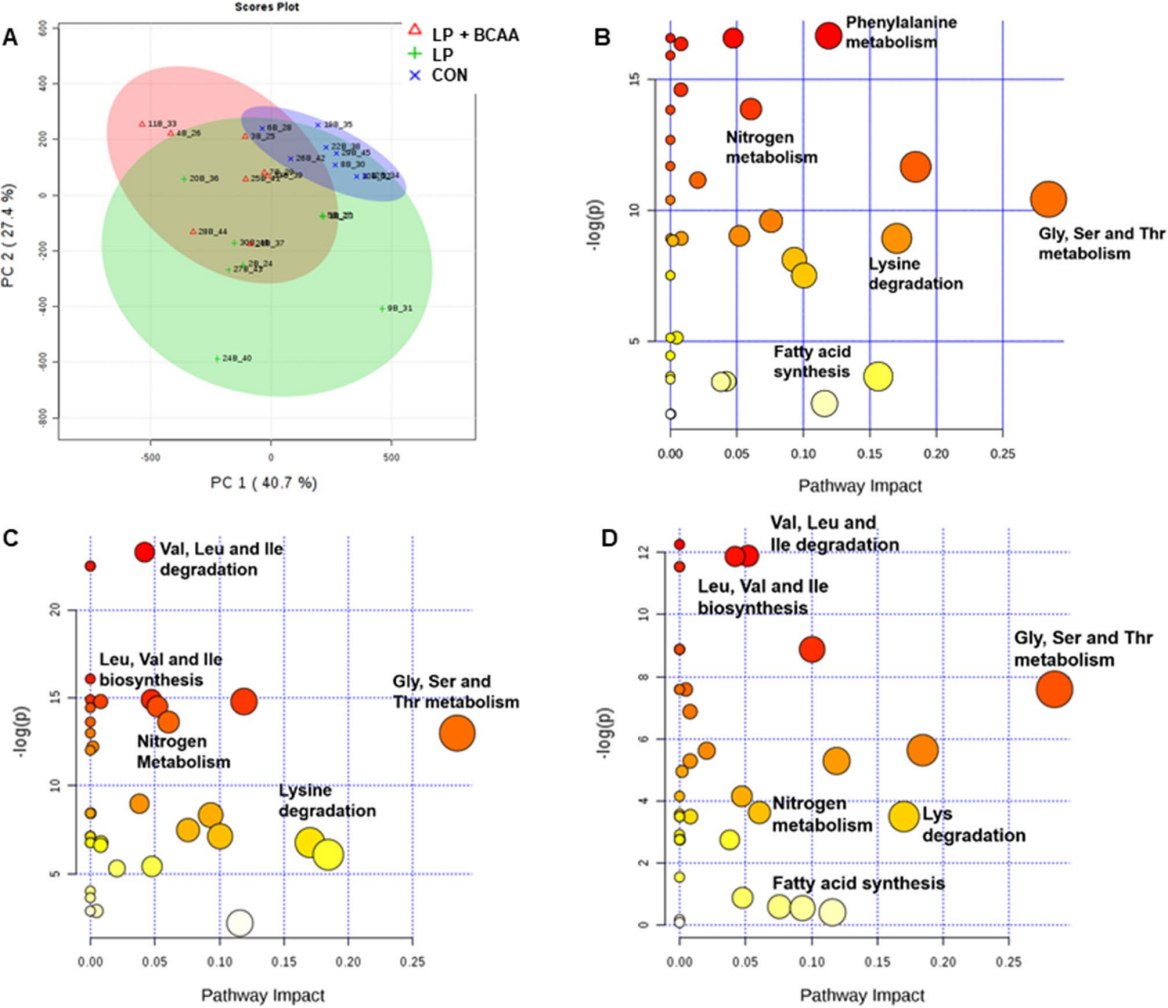
Principal component analysis (PCA) score plots and the pathway analysis map of plasma metabolites in pigs fed with very low protein diets supplemented with branched-chain amino acids. (**A**) PCA score plot for plasma metabolites. Individual pigs are shown as nodes, (**B**, **C**, **D**) the map of pathway analysis for the plasma. Each circle is obtained from topology analysis representing a metabolic pathway with the scores. The x-axis denotes the pathway impact and the y-axis represents the pathway enrichment. The size of each circle is based on its impact value while the color of each circle is based on its *P* value. Larger size circles have higher pathway impact, while darker color circles are indicative of more significant changes of metabolites and pathway enrichment. CON, control diet; LP, low protein diet; LP + BCAA, low protein diet supplemented with branched-chain amino acids. (**B**) CON vs. LP + BCAA, (**C**) CON vs. LP, (**D**) LP vs. LP + BCAA. n = 8.

The principal component analysis (PCA) score plot displays a clear separation between LP + BCAA and CON as well as LP and CON for plasma metabolites (Fig. 3A). No clear separation for plasma metabolites was seen between LP and LP + BCAA (Fig. 3A). There was a positive loading for CON and negative loading for LP + BCAA on the PC 1 axis. Also, there was a positive loading for CON and primarily negative loading for LP on the PC 2 axis. The PC 1 explains 40.7% of variation of metabolite changes within samples and PC 2 is indicative of 27.4% of the variation. The metabolic pathway enrichment analysis showed that nitrogen metabolism, lysine degradation, and glycine, serine and threonine degradation is influenced when dietary treatments were compared in two by two comparisons (Fig. 3). Comparing the CON and LP + BCAA groups, phenylalanine metabolism and fatty acid synthesis pathways were highly affected (Fig. 3B), while valine, leucine and isoleucine degradation and biosynthesis were influenced when CON was compared to LP (Fig. 3C). When LP was compared with LP + BCAA, valine, leucine and isoleucine degradation and biosynthesis as well as fatty acids synthesis pathways were changed (Fig. 3D).

As shown in Table 1, the metabolites involved in nitrogen or protein metabolism were influenced with dietary groups. Hydroxylamine was higher for LP compared to the CON with no differences between LP + BCAA and CON. Urea and phenylethylamine were significantly lower for LP + BCAA compared to CON. As expected, dietary groups influenced the AA profile (Table 1). Compared to CON and LP + BCAA, the LP had a lower abundance of isoleucine and valine. Interestingly, LP + BCAA had higher valine compared to CON. Relative to CON, the LP had the highest amount of threonine and methionine among groups, while their amount for LP + BCAA was higher than CON. Compared to CON, the LP + BCAA had the highest amount of glycine among groups, while its amount for LP was higher than CON. LP and LP + BCAA had a lower abundance of tyrosine than CON. Among dietary treatments, LP + BCAA had the highest abundance of oxoproline. Also, AA metabolism metabolites were significantly affected by dietary treatments (Table 1). Interestingly, plasma aminomalonate, a metabolite involved in AA metabolism, was significantly higher in LP + BCAA compared to both CON and LP. Kynurenic acid and 2-aminobutyric acid, also involved in AA metabolism, were higher in both LP and LP + BCAA relative to CON. 2-ketoisocaproic acid was lower in LP compared to both CON and LP + BCAA. The metabolites involved in BCAA and microbiome metabolism including indole-3-acetate and indole-3-lactate were greater in LP + BCAA compared to CON. The 5-aminovaleric acid and indole-3-propionic acid were higher in LP relative to CON and LP + BCAA.

**Table 1:** Effect of very low protein diets supplemented with branched-chain amino acids on blood metabolomics profile.

Metabolites	CON^1^	LP^1^	LP + BCAA^1^	SEM^2^	*P* value
**Microbiome metabolism**					
Indole-3-acetate	1263.01^a^	3547.13^b^	2150.38^b^	299.05	0.001
Indole-3-lactate	2661.38^a^	6937.63^ab^	8215.88^b^	822.43	0.001
5-aminovaleric acid	3197.50^a^	9937.38^b^	4806.25^a^	963.34	0.004
Phenylethylamine	4743.25^a^	2978.38^ab^	1226.50^b^	465.3	0.009
Indole-3-propionic acid	2513.13^a^	7533.13^b^	5072.13^ab^	791.47	0.011
**Carbohydrate derivatives**					
Conduritol-beta-epoxide	15,871.75^a^	2909.88^b^	3352.38^b^	1344.03	< 0.001
Pinitol	8378.63^a^	1652.00^b^	1489.75^b^	756.54	< 0.001
Lactobionic acid	2877.88^a^	529.38^b^	590.88^b^	275.50	< 0.001
*N*-acetylornithine	7457.88^a^	2728.88^b^	1251.88^b^	654.25	< 0.001
Ribonic acid	2162.63^a^	3657.25^b^	2626.75^a^	233.95	< 0.001
Arabitol	28,118.38^a^	71,562.50^b^	37,037.00^a^	6561.00	0.002
Saccharic acid	2743.50^a^	1517.38^b^	1383.75^b^	159.84	0.001
**Carbohydrate metabolism**					
Uridine	1234.38^a^	515.63^b^	749.75^a^	82.46	0.001
Glucose-6-phosphate	1206.38^a^	441.13^b^	814.88^b^	99.26	0.007
**Carbohydrates**					
Beta-gentiobiose	2329.13^a^	600.63^b^	563.88^b^	216.93	< 0.001
Ketohexose	209.00	213.88	205.00	12.77	< 0.001
Raffinose	980.37^a^	345.50^b^	333.38^b^	85.78	0.001
1,5-anydroglucitol	3301.25^a^	6019.63^b^	3342.75^a^	505.60	0.009
**Protein metabolism**					
Urea	940,405.80^a^	635,158.50^b^	293,551.30^c^	66,744.59	< 0.001
Hydroxylamine	45,753.00^b^	52,395.38^a^	49,788.75^ab^	2094.84	0.010
**Amino acids**					
Tyrosine	399,911.30^a^	120,268.5^b^	87,610.89^b^	30,930.61	< 0.001
Isoleucine	344,809.10^a^	92,163.25^b^	325,098.50^a^	26,711.03	< 0.001
Phenylalanine	110,364.60^a^	55,063.13^b^	31,340.13^b^	7767.45	< 0.001
Valine	407,796.30^a^	84,820.75^b^	602,500.30^c^	51,100.37	< 0.001
Threonine	121,075.50^a^	1,050,724.00^b^	435,425.50^c^	92,325.20	< 0.001
Glycine	342,278.90^a^	509,391.50^b^	674,927.90^c^	36,520.40	< 0.001
Ornithine	228,654.00^a^	104,584.90^b^	82,086.00^b^	15,020.05	< 0.001
Oxoproline	462,381.90^a^	39,389.90^a^	582,909.60^b^	26,933.36	< 0.001
Methionine	39,023.25^a^	74,093.13^b^	56,962.88^c^	4211.50	< 0.001
Citrulline	17,085.63^a^	8382.00^b^	6267.88^b^	1326.06	0.009
**Fatty acid metabolites**					
2,3-dihydroxybutanoic acid	404.13^a^	3138.88^b^	1388.63^c^	259.35	< 0.001
2-hydroxyvaleric acid	2348.38^a^	1634.75^a^	4324.25^b^	289.61	< 0.001
3-hydroxybutyric acid	5419.25^a^	3482.88^a^	9183.5^b^	661.57	< 0.001
Isohexonic acid	3362.13^a^	1278.38^b^	1298.88^b^	228.72	< 0.001
**Amino acids metabolism metabolites**					
2-ketoisocaproic acid	21,596.50^a^	10,746.13^b^	23,537.63^a^	1466.81	< 0.001
Aminomalonate	41,156.63^a^	52,539.88^a^	59,765.13^b^	2894.44	< 0.001
2-aminobutyric acid	11,633.13^a^	41,811.25^b^	39,925.88^b^	3525.16	< 0.001
Kynurenic acid	403.25^a^	690.13^b^	690.13^b^	56.56	0.002
Glutaric acid	200.38^a^	312.13^ab^	325.00^a^	22.60	0.002
**Amino acid derivatives**					
Methionine sulfoxide	22,322.13^a^	30,949.88^b^	28,557.00^b^	1555.36	0.002
**Vitamins**					
Tocopherol alpha	3098.50^a^	4690.25^b^	4112.88^b^	232.57	< 0.001

^1^CON, control diet; LP, low protein diet; LP + BCAA, low protein diet supplemented with branched-chain amino acids. The values are mean peak height. n = 8.

^2^SEM, standard errors of means.

^a,b,c^ Within a row, values with different superscripts are different (*P ≤ *0.05).

Carbohydrate metabolites including beta-gentiobiose, and raffinose were lower in both LP and LP + BCAA compared to CON (Table 1). The peak height of 1,5 anydroglucitol was higher for LP than CON and LP + BCAA. The metabolites involved in carbohydrate metabolism, such as uridine and glucose-6-phosphate, were significantly lower in LP compared to CON and LP + BCAA. The pathways analysis indicated that fatty acids synthesis pathways were influenced by dietary treatments. In this study, 2-hydroxyvaleric acid and 3-hydroxybutyric acid, fatty acid metabolites, were higher in the LP + BCAA diet compared to LP and CON. The concentrations of isohexonic acid were lower in both LP + BCAA and LP, whereas the amount of 2,3-dihydroxybutanoic acid was greater in these two groups compared to CON.

### Microbiota

**Figure 4:**
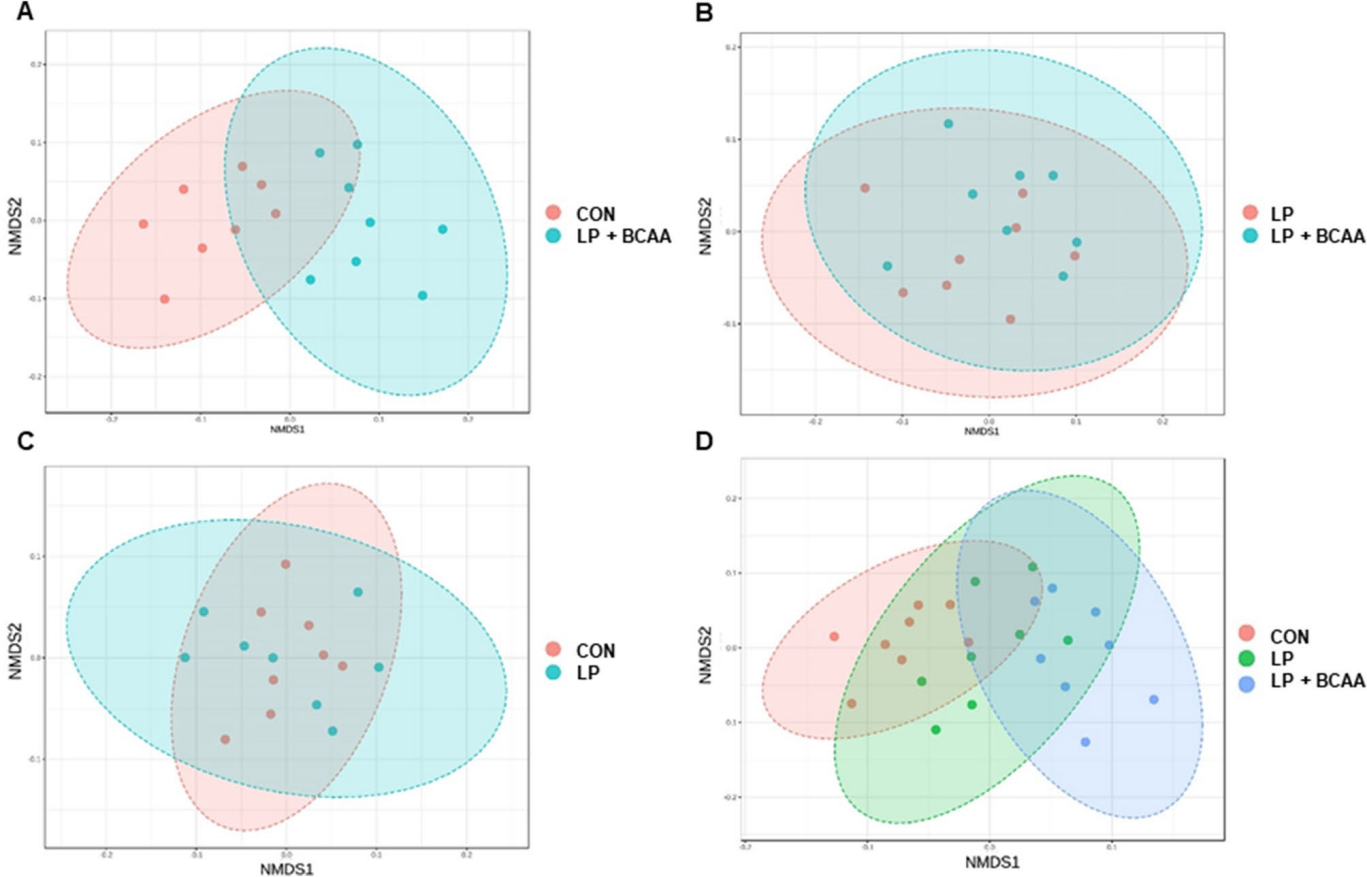
Beta diversity of the fecal bacterial community in pigs fed with very low protein diets supplemented with branched-chain amino acids. Non-metric multidimensional scaling (NMDS) of fecal microbiota for (**A**) CON vs. LP + BCAA, (**B**) LP vs. LP + BCAA, (**C**) CON vs. LP, (**D**) CON vs. LP vs. LP + BCAA. Pigs are grouped based on their dietary treatments, *i.e.* CON, control diet; LP, low protein diet; LP + BCAA, low protein diet supplemented with branched-chain amino acids. Each node represents an individual pig. Differences were considered significant at *P* < 0.05. The Permutational Multivariate Analysis of Variance (PERMANOVA) *P* values for CON vs. LP + BCAA, LP vs. LP + BCAA, CON vs. LP and CON vs. LP vs. LP + BCAA were < 0.004, < 0.197, 0.178 and < 0.016 respectively. n = 8.

**Figure 5:**
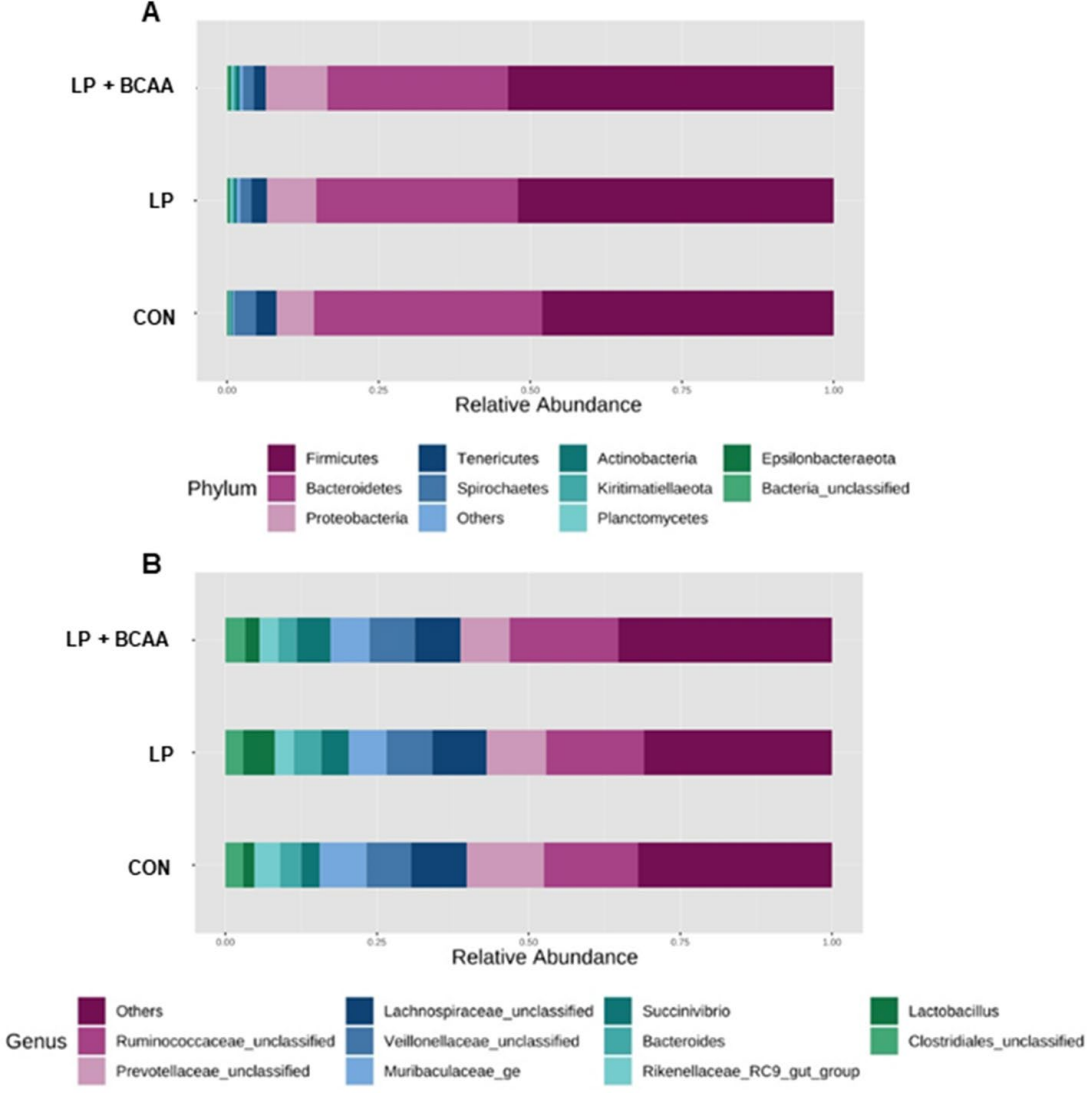
The effect of very low protein diets supplemented with branched-chain amino acids on fecal bacterial community at phylum and genus levels. (**A**) The relative abundance of bacterial community composition at phylum level in fecal samples of pigs fed with very low protein diets supplemented with branched-chain amino acids. Only the top 10 phyla are depicted for clarity. (**B**) The relative abundance of bacterial community composition at genus level in fecal samples of pigs fed with very low protein diets supplemented with branched-chain amino acids. Only the top 10 genera are depicted for clarity. CON, control diet; LP, low protein diet; LP + BCAA, low protein diet supplemented with branched-chain amino acids. n = 8.

The rarefaction curve analysis demonstrated that the species richness of all analyzed samples reached a stable plateau at 40,000 reads per sample (Supplementary Fig. S2), which is suggestive of the sufficient sequencing depth to saturate the bacterial communities in fecal samples. Beta diversity of the fecal bacterial community among dietary treatments is shown in Fig. 4. The Nonmetric Multidimensional Scaling (NMDS) showed some significant separation and clustering when CON vs. LP + BCAA (Permutational Multivariate Analysis of Variance [PERMANOVA] *P* value < 0.004; Fig. 4A) and CON vs. LP vs. LP + BCAA (PERMANOVA *P* value < 0.016; Fig. 4D) were considered suggestive of the differences in gut microbiota composition among pigs fed these diets. Fecal bacterial composition of LP vs. LP + BCAA and CON vs. LP showed no clear clustering (PERMANOVA analysis *P* values < 0.197 and 0.178, respectively; Fig. 4B,C). Alpha diversity of gut bacterial community in each sample is shown in Supplementary Fig. S3. Shannon diversity, which relates both operational taxonomic units (OTU) richness and evenness, tended to be significantly different among dietary groups (*P* value = 0.075; Supplementary Fig. S3C). Statistical analysis showed no difference for Chao1, Observed and Simpson indices (*P* values = 0.638, 0.153 and 0.120, respectively; Supplementary Fig. S3A, B, D). Overall, the three main phylum present among all dietary treatments were Firmicutes, Bacteriodetes and Proteobacteria (Fig. 5A and Supplementary Fig. S4A). At genus level, *Ruminococcaceae-unclassified*, *Prevotellaceae-unclassified, Lachnospiraceae-unclassified* and *Veillonellaceae-unclassified* were the most abundant communities for all diets (Fig. 5B and Supplementary Fig. S4B).

**Figure 6:**
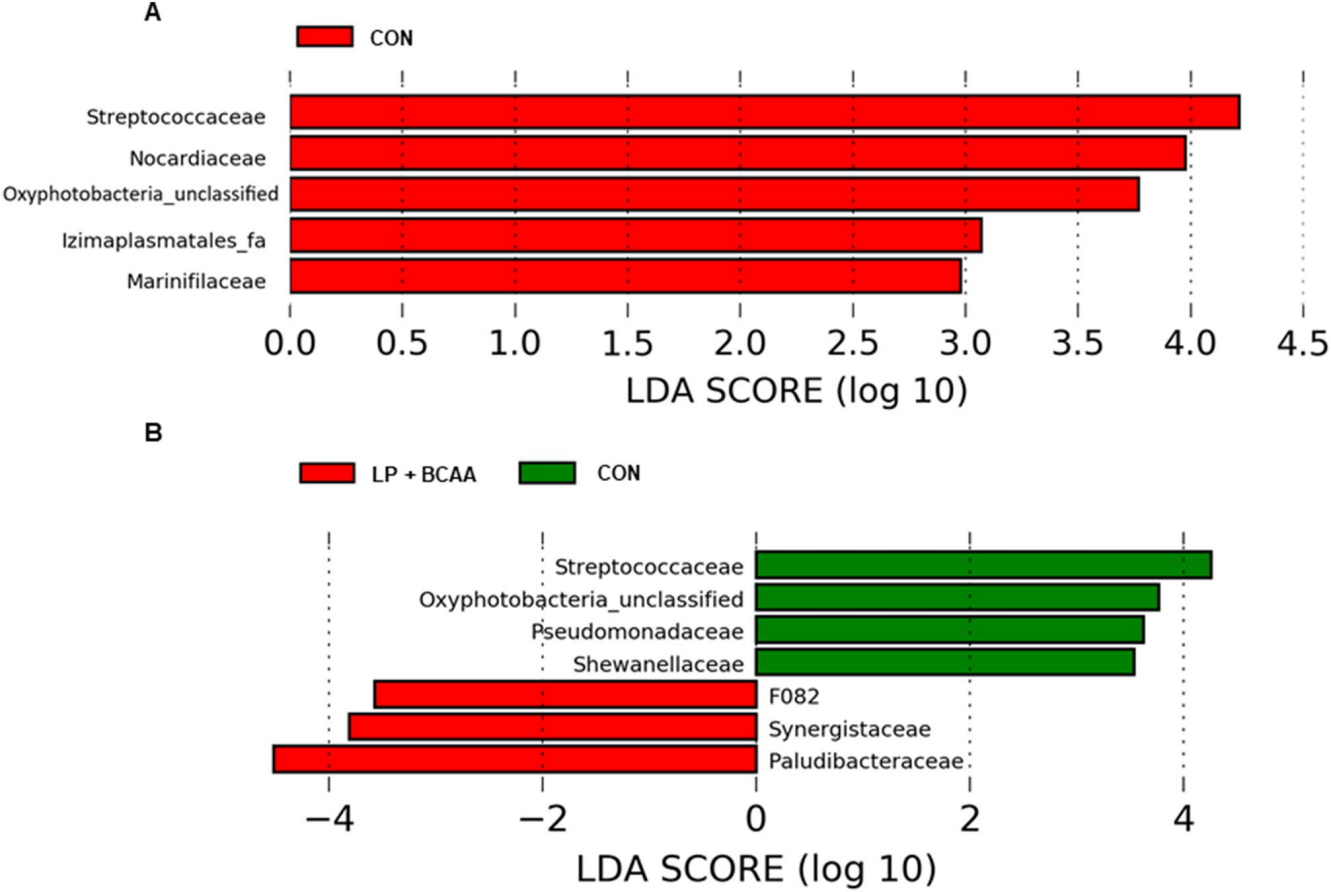
Effect of very low protein diets supplemented with branched-chain amino acids on fecal microbiota composition at family level using linear discriminant analysis (LDA) with effect size measurements (LEfSe). (**A**) CON, control diet vs. LP, low protein diet. (**B**) CON vs. LP + BCAA, low protein diet supplemented with branched-chain amino acids. No differences in LDA analysis were seen for LP vs. LP + BCAA. n = 8.

Linear discriminant analysis (LDA) with effect size measurements (LEfSe) was used to determine the most notable bacterial communities at family level between dietary treatments. Compared to LP, fecal samples of CON pigs had more proportions of *Streptococcaceae, Nocardiaceae, Oxophotobacteria_unclassified, Izimaplasmatales_fa* and *Marinifilaceae* (LDA [log_10_] score, > 2.0; Fig. 6A and Supplementary Fig. S5). The feces of pigs fed with CON diet contained higher proportions of *Streptococcaceae, Oxyphotobacteria_unclassified, Pseudomonadaceae* and *Shewanellaceae* (LDA [log10] score, > 2.0), compared to LP + BCAA (Fig. 6B and Supplementary Fig. S6), while, the feces of LP + BCAA pigs were more enriched in *Paludibacteraceae, Synergistaceae and F082* (LDA [log_10_] score, > 2.0; Fig. 6B). There were no significant differences in fecal bacterial communities of pigs fed with LP in comparison with those received LP + BCAA diet.

## Discussion

Very low protein diets (> 25% reduced CP) may be used for reducing weaning stress and its associated complications, feed cost and nitrogen excretion in young pigs. However, these diets depress the growth efficiency^3,14–16^ and may not be practical for commercial swine production. In the light of our recent findings^33^ showing that BCAA degradation and biosynthesis pathways are greatly influenced and these AA and their metabolites and metabolism intermediates are decreased in circulation when pigs are fed with VLP diets, we hypothesized that supplementing these diets with BCAA may improve the growth of nursery pigs. Little is understood on the effect of VLP diets supplemented with BCAA on growth performance and energy balance. Further, there is no published research on the gut microbiota composition and blood metabolomics profile of pigs fed with VLP diets supplemented with BCAA. Therefore, the objective of this study was to assess the effect of VLP diets fortified with BCAA on growth performance, energy balance (*i.e.* feed intake and energy expenditure), blood metabolomics profile and gut microbiota composition. Our study revealed several important findings: (1) reducing the dietary CP (*i.e.* LP) decreased the FI and BW, but supplementation of LP with BCAA (*i.e.* LP + BCAA) improved the overall FI calculated for the whole study period, tended to increase the overall ADG, increased the EE and RQ transiently and delayed the FI and BW depression for ~ 2 weeks, indicative of the short-term protective role of BCAA against growth performance depression when the total protein is dropped, (2) decreasing dietary CP (*i.e.* LP) greatly influenced the degradation and biosynthesis of leucine, isoleucine and valine, but supplementation of LP with BCAA (*i.e.* LP + BCAA) specifically affected the phenylalanine metabolism and fatty acids synthesis pathways. In particular, the abundances of 2-hydroxyvaleric and 3-hydroxybutyric acids were increased in plasma of pigs fed with LP + BCAA, (3) nitrogen or protein metabolism were influenced by dietary groups with the lowest concentration of blood urea for pigs fed with LP + BCAA and of citrulline and ornithine for both LP and LP + BCAA groups, (4) compared to control animals, the pigs offered with LP + BCAA diet had higher abundance of *Paludibacteraceae* and *Synergistaceae* in their feces, while that was less enriched in *Streptococcaceae, Oxyphotobacteria_unclassified, Pseudomonadaceae and Shewanellaceae*. This is suggestive of better health and carbohydrates digestion capacity in pigs fed LP + BCAA. Thus, these data show that supplementing VLP diets with BCAA delays the negative effects of these diets on growth performance, which is related with alterations in energy balance, blood metabolomics profile and fecal microbiota composition.

Reducing the dietary CP significantly reduced BW and G:F. This is consistent with previous findings showing that VLP diets supplemented with or without first few limiting AA do not improve the growth performance^3,14–16,33^. This can be due to reduced FI, increased EE and altered metabolism of BCAA and gut microbiota composition in pigs fed with VLP diets^33^. Although previous research has provided evidence on beneficial effects of supplemental BCAA on growth of nursery and growing pigs fed with diets with normal^31,32^ or slightly low protein content^19,22,23,25–30,39^ diets, little is understood whether supplementing VLP diets with BCAA can partially or completely recover the VLP induced depression of growth performance. In the current study, supplementing VLP diets with BCAA at levels recommended by Nutrient Requirements of Swine^8^ delayed the onset of reduction in BW in nursery pigs. In another study, supplementing a mixture of BCAA, histidine and phenylalanine to VLP diets (12 and 14% CP) improved the growth performance of nursery pigs^34^. The growth performance of pigs fed with VLP diets supplemented with BCAA was similar to CON group during early weeks of study, which is likely due to lack of difference in FI between these two groups during first two weeks of study. In line with our findings in stimulatory effects of supplemental BCAA on FI, previous studies have shown that dietary supplementation of BCAA to slightly low protein diets^23,24^ or a combination of BCAA, histidine and phenylalanine to VLP diets^34^ recovered the reduced FI and growth in weanling pigs. These data suggest a short-term protective role of BCAA against reduction in growth performance. To the best our knowledge, our study is the first reporting a temporary increase of EE and RQ in pigs fed with LP + BCAA. Further research is warranted to understand the impact of VLP diets with or without supplemental BCAA on EE in pigs.

There are no published data on blood metabolomics profile of pigs fed with VLP diets supplemented with BCAA. Here for the first time we report that supplementation of VLP diets with BCAA affected the phenylalanine metabolism and fatty acids synthesis pathways. Pigs fed with LP + BCAA diet had higher plasma 2-hydroxyvaleric acid and 3-hydroxybutyric acid, which are produced from BCAA catabolism^40^. Similarly, others found that BCAA contributed a great proportion to the production of fatty acids. Specifically, leucine and isoleucine contribute to lipogenic acetyl-CoA pool, and valine and isoleucine contributed to lipogenic propionyl-CoA pool in adipocytes^41^. The increase in plasma 2-hydroxyvaleric acid and 3-hydroxybutric acid is due to increased influx of BCAA into organs such as skeletal muscle and liver that are involved in degradation and metabolism of BCAA. Further, the metabolic pathway analysis of plasma metabolites revealed that nitrogen metabolism pathway was significantly influenced by VLP diets supplemented with or without BCAA. The plasma urea concentration was decreased in LP + BCAA and LP groups with the lowest value for LP + BCAA. Likewise, other studies showed that feeding pigs with protein-restricted diets reduced the plasma urea concentration^7,16,22,42^. Moreover, others found that supplementing protein-restricted diet with BCAA further increased the nitrogen retention and decreased plasma urea concentration in weaned pigs compared to those fed with protein-restricted diet^22^. BCAA supplementation has been suggested to be beneficial for dietary nitrogen utilization and reducing the nitrogen excretion^8^ likely through improving the dietary AA balance. In addition to urea, plasma citrulline and ornithine, two intermediates in the urea cycle, were decreased in both LP and LP + BCAA groups. Further studies are warranted to examine the nitrogen excretion through feces and urine and nitrogen metabolism at tissues (e.g. kidney) level in pigs receiving low protein diets supplemented with BCAA. Analyzing the fecal, urine and tissues for nitrogen excretion and metabolism will allow to understand whether severe reduction of CP and further supplementation with BCAA, facilitates the efficient nitrogen utilization, improves the nitrogen retention and reduces the nitrogen excretion to the environment.

In the current study, in concordance with our previous finding^33^, feeding pigs with VLP diets without supplemental BCAA greatly influenced the degradation and biosynthesis of leucine, isoleucine and valine. The amount of plasma isoleucine and valine was higher in LP + BCAA pigs compared to LP group. Other studies have similarly shown that dietary supplementation of BCAA increases the plasma concentration of these AA^22,43^. Our data are in agreement with others^22^ showing that the plasma valine in LP + BCAA group was even higher than CON. LP + BCAA had higher amounts of 2-ketoisocaproic acid, an intermediate of leucine metabolism compared to LP. Further, similar to others, the concentration of threonine and methionine was higher in both LP and LP + BCAA than CON^22^, with the greatest concentration of these two AA for LP group. The lower concentration of threonine and methionine in LP + BCAA over LP group may be due to competition of these AA with BCAA for absorption in the gut. These data suggest that the levels of BCAA added to diets should be optimized in relation with other limiting AA. The partial improvement in FI and growth performance in LP + BCAA in the current study might be due improved plasma profile of supplemented BCAA as well as methionine and threonine. The lack of improvement in all plasma essential AA such as tyrosine and phenylalanine may contribute to partial reversal, but not complete improvement of reduced FI and growth of pigs fed with VLP diets following BCAA supplementation. Further research is needed to characterize the ideal combination of supplemental limiting AA with BCAA that can significantly annul the VLP induced depression of growth performance.

There is paucity of data on gut microbiota composition of pigs fed with VLP diets supplemented with BCAA. In the present study, the main three phyla in the feces of all dietary treatments where Firmicutes, Bacteriodetes and Proteobacteria*.* This is in line with our previous study where Firmicutes, Bacteriodetes and Proteobacteria were the primary phyla in the feces of pigs fed with diets with variable protein contents^33^. Similarly, others showed that Firmicutes and Bacteriodetes contribute to the majority of phyla in feces and cecal contents of pre and post weaned and growing pigs^44–46^. At family level, *Ruminococcaceae, Prevotellaceae, Lachnospiraceae* and *Veillonellaceae* were the most dominant bacterial communities in the feces of pigs assigned to all dietary groups. In concordance with these data, *Prevotellaceae, Veillonellaceae, Ruminococcaceae*, and *Lachnospiraceae* were previously reported as the most dominant communities at family level in the feces of pigs fed 12–24% CP^33^. Likewise, others reported a high abundance of *Prevotellaceae, Ruminococcaceae and Lactobacillaceae* in the feces of nursery pigs^47^. Increased abundance of *Prevotellaceae* and *Ruminococcaceae* among all groups, which are involved in digestion of polysaccharides may be due to availability of plant-based substrates for their action^44,48–51^.

LDA for fecal microbiota showed that pigs fed with LP + BCAA and LP had lower proportions of *Streptococcaceae*, compared to CON. Similarly, others showed that *Streptococcaceae* abundance in ileal contents is decreased with reducing the protein concentration^52^. Since *Streptococcaceae* is a predominant AA utilizer^53,54^, the drop of this family in low protein fed pigs could be the result of protein shortage as a substrate for fermentation. Some members of *Streptococcaceae* family such as *Streptococcus* are involved in developing infectious diseases^55^. Therefore, reduced *Streptococcaceae* family in feces of pigs fed with VLP diets might be beneficial for their health. Other communities such as *Oxyphotobacteria*, *Pseudomonadaceae* and *Shewanellaceae*, which were also found to be less enriched in feces of LP + BCAA pigs have been identified among gut microflora populations in animals and insects^56–58^ while further investigation is required to understand their role as part of swine gut microbiome. The pigs offered with LP + BCAA diet had higher abundance of *Paludibacteraceae and Synergistaceae* in their feces, which given the low concentration of protein in this diet, these bacteria are unlikely involved in protein degradation. Some members of *Paludibacteraceae,* such as *Paludibacter* have been identified in the swine gut content^59^. *Paludibacter* can utilize various soluble carbohydrates and simple sugars and produce acetate and propionate^60^. The increased population of *Paludibacteraceae* in LP + BCAA may be due to high concentration of carbohydrates in this diet that can be used as a substrate for this family. The phylum Synergistetes, to which *Synergistaceae* belongs, is one of the rare populations in the swine gut^61^ and the role of this phylum in the gut is not completely known^62^. *Synergistaceae* has been regarded as one of the major microbial communities in the gut of healthy koala^63^ and the members of this family has been shown to be involved in degradation of toxic pyridinediols in the animals’ diet^64^ and plant toxin fluoroacetate^65^. Overall, these data suggest that LP + BCAA may optimize the gut microbial population by decreasing the *Streptococcaceae* and increasing the *Paludibacteraceae and Synergistaceae*. Whether these specific alterations in gut microbiota composition contribute to BCAA-induced improvement in growth performance of pigs fed with VLP diets, it is still remained to be determined.

## Conclusions

Feeding nursery pigs with very low protein diets resulted in decreased feed intake, body weight and gain:feed, but supplementing these diets with branched-chain amino acids improved the overall feed intake calculated for the entire study period and delayed the feed intake and body weight depression for ~ 2 weeks. Supplementation of branched chain amino acid to very low protein diets produced differential responses in plasma metabolomics profile and feces microbial composition. Very low protein diets influenced the metabolism of branched-chain amino acids, but when supplemented with dietary branched chain amino acids, mainly the fatty acids synthesis and nitrogen and protein metabolism pathways were impacted. The feces of pigs fed with very low protein diets supplemented with branched-chain amino acids had higher proportion of *Paludibacteraceae* and *Synergistaceae*, but was less enriched in *Streptococcaceae, Oxyphotobacteria_unclassified, Pseudomonadaceae and Shewanellaceae*. Thus, supplemental branched-chain amino acids temporarily restores the negative effects of very low protein diets on growth performance in weaned pigs, which is associated with changes in energy balance, blood metabolomics profile and fecal microbiota composition.

## Methods

### Animals, housing and diets

All methods performed in this study was in accordance with the guidelines and regulations of Institutional Animal Care and Use Committee (IACUC) at Oklahoma State University. The experimental procedures for this study were approved by the Oklahoma State University IACUC (Animal Care and Use Protocol – AG-17–27). A total of 24 crossbred (PIC) weaned pigs (3 wk old) were housed in a facility with controlled temperature and ventilation following the animal husbandry procedures described in our previous publication^33^. Pigs were adapted for 1 wk with a common pelleted nursery starter diet (United Animal Health, Sheridan, IN) prior to staring the experiment. Following the adaption period, the pigs were weight-matched (8.26 ± 0.34 kg), individually housed and randomly assigned into three dietary treatments (n = 8) including: CON, LP and LP + BCAA for a period of 4 wk. The ingredients and composition of the experimental diets are shown in Table 2. The CON was a positive control diet with standard protein content and was formulated based on Nutrient Requirements of Swine^8^ standard ileal digestibility (SID) recommendation for AA, which became to be equivalent to ~ 22% CP as we previously described^33^. The CP contents of LP and LP + BCAA diets were dropped by reducing the soybean meal and their CP was kept as consistent as possible to examine whether the growth and energy balance of LP group can be restored by supplementing this diet with adding BCAA. The LP was a negative control diet supplemented with limiting AA (*i.e.* lysine, methionine, threonine and tryptophan) with ~ 13% CP while LP + BCAA was a negative control supplemented with a mixture of limiting AA and BCAA with ~ 14% CP. Supplemented limiting AA and BCAA to LP and LP + BCAA groups, were in equivalent amounts to the CON. Along with changes in soybean meal content to achieve the desired protein content, the corn and cornstarch amounts as sources of carbohydrate were manipulated in order to attain consistent energy content among all dietary treatments. All diets were isocaloric. Nutrient Requirements of Swine^8^ recommends formulating three diets for three phases during 6 weeks of nursery period based on pigs’ body weight range mainly for the purpose of efficient swine production. However, in the current study no growth phase feeding was applied and diets were formulated to meet the requirements of 7–11 kg pigs^8^ to avoid the variations induced by multiple diets offered throughout the experiment. The pigs had ad libitum access to feed and water, throughout the study. Animals were fed once a day at ~ 1500.

**Table 2:** Composition of experimental diets (as-fed basis).

Ingredients, %	CON^1^	LP^1^	LP + BCAA^1^
Corn, yellow dent	52.60	76.21	76.09
Fish meal, menhaden	5.00	5.01	5.00
Soybean meal, 47.5% CP	34.50	3.41	3.40
Whey, dried	6.00	6.01	6.00
Corn starch	–	4.43	2.60
Dicalcium phosphate 18.5%	0.65	1.45	1.45
Limestone	0.59	0.45	0.45
Salt	0.26	0.30	0.30
Vitamin Premix^2^	0.25	0.30	0.30
Mineral Premix^3^	0.15	0.15	0.15
Vitamin E^4^	–	0.04	0.04
Lysine, sulfate^5^	–	1.46	1.46
DL-Methionine^5^	–	0.15	0.15
L-Threonine^5^	–	0.45	0.45
L-Trytophan^5^	–	0.18	0.18
L-Isoleucine^5^	–	–	0.55
L-Valine^5^	–	–	0.57
L-Leucine^5^	–	–	0.82
**Chemical composition**			
Calculated crude protein, %^6^	24.59	13.52	14.82
Analyzed crude protein, %	22.10	13.30	14.30
SID Lysine, %^6^	1.32	1.32	1.32
SID Threonine, %^6^	0.83	0.83	0.83
SID Methionine, %^6^	0.38	0.38	0.38
SID Tryptophan, %^6^	0.27	0.27	0.27
SID Leucine, %^6^	1.87	1.08	1.88
SID Isoleucine, %^6^	0.94	0.40	0.94
SID Valine, %^6^	1.04	0.50	1.04
Calculated crude fat, %^6^	3.61	3.60	3.59
Analyzed crude fat, %	2.20	2.50	2.80
ME, Mcal/kg^6^	3.32	3.36	3.34

^1^CON, control diet; LP, low protein diet; LP + BCAA, low protein diet supplemented with branched-chain amino acids.

^2^Vitamin Premix: vitamin A, 1,650,000 IU/kg; vitamin D3, 660,000 IU/kg; vitamin E, 17,600 IU/kg; menadione, 1,320 mg/kg; vitamin B_12_, 13.2 mg/kg; niacin, 19,800 mg/kg; D-pantothenic acid, 11,000 mg/kg; riboflavin, 3,300 mg/kg; phytase, 299,376 FYT/kg.

^3^Mineral Premix: copper, 11,000 ppm; iodine, 198 ppm; iron, 73,000 ppm; manganese, 22,000; selenium, 198 ppm; zinc, 73,000 ppm.

^4^Vitamin E: 44,444 IU/kg.

^5^Contains: 54.6% L-Lysine, 99.0% DL-Methionine, 98.5% L-Threonine, 98.0% L-Tryptophan, 98.0% L-Isoleucine, 96.5% L-Valine, 98.5% L-Leucine.

^6^Values were calculated using National Swine Nutrition Guide software (NSNG, V 2.0); standard ileal digestibility (SID); metabolizable energy (ME).

### Metabolic measurements

Individual FI and BW were measured daily and weekly, respectively, throughout the experiment. Overall ADG, ADFI, ADPI, G:F, G:P and weekly FI, BW, G:F and G:P were then calculated. An indirect calorimetry system (AEI Technologies, Chicago, IL) was used to measure the daily EE and RQ. The pigs from all three dietary groups were rotated in metabolic chambers (total 6 chambers) for measuring the EE in 48 h cycles during the entire study. The pigs had free access to their experimental diet and water in each metabolic chamber. Prior to starting daily EE measurements, the O_2_ and CO_2_ sensors were calibrated by known volume of O_2_ (16% and 21%) and CO_2_ (0.03% and 4%). The EE was measured from 0800 to 1500 h. To allow the system to stabilize, the first 2 h data were excluded from statistical analysis. The flow rate was set at 4–20 L/min depending upon the weight of pigs with a sampling time of 5 s/chamber, stabilization period of 55 s and a reference air measurement after every 3 chambers. The O_2_ consumption rate (VO_2_ ml/min), CO_2_ production rate (VCO_2_ ml/min) and the RQ (VCO_2_/VO_2_) were measured. The normalized EE [(kcal/h)/kg BW^0.75^] was calculated as follows: [3.815 + (1.232 × RQ)] × VO2 (L/h)^33,66,67^^.^

### Feed, fecal and blood sample collection

A subsample of feed was collected from each feed bag (~ 50 g) and pooled for each diet during the diet preparations. The feed samples were stored at -20 °C until further analysis. Fresh fecal samples were collected in pre-labeled 50 mL falcon tubes (VWR Radnor, PA) from the rectum of pigs in all groups on wk 4 of the study. The samples were placed on ice, transferred to the lab immediately and stored at -80 °C until later analysis. Blood samples were collected on wk 4 of the study. About 10 ml blood was taken from the jugular anterior vena cava of each pig in a 10 mL EDTA coated tube (Vacutainer BD, Franklin Lakes, NJ). The blood samples were placed on ice, transferred to the lab, centrifuged (2000×*g* for 10 min at 4 °C) and plasma was separated. The collected plasma was stored at -80 °C until further analysis.

### Diets composition analysis

The feed samples were analyzed by Servi-Tech laboratories (Dodge City, KS) for dry matter, CP, crude fat, crude fiber, calcium, phosphorus and nitrogen as previously described^68–73^.

### Plasma cytokines analysis

Plasma samples were analyzed in duplicate for tumor necrosis factor-α (TNF-α), interleukin 6 (IL-6) and interleukin 12p40 (IL-12p40) using an enzyme-linked immunosorbent assay (R&D Systems, Inc., Minneapolis, MN) and ProcartaPlex multiplex immunoassay kits (ThermoFisher Scientific, Inc., Waltham, MA). Samples were analyzed according to the manufacturer’s instructions. The absorbance values were measured using a microplate reader (Spectramax M3; Molecular Devices, LLC, San Jose, CA) at 450 nm with setting the correction wavelength at 570 nm. The cytokines concentration was quantified after generating the standard curve with samples with known concentrations. The intraassay CV for TNF-α was 4.4%, IL-6 was 16.5% and IL-12p40 was 7.4%.

### Plasma metabolomics

Plasma metabolomics profile was analyzed at West Coast Metabolomics Center (UC Davis, Davis, CA) following the established protocols^74,75^ as we previously described^33^. Briefly, following sample preparation for removal of protein from plasma and derivatization, the samples were analyzed by gas chromatography (GC)- mass spectrometry (MS) using a time of flight (TOF) mass spectrometer (Leco Pegasus IV). A GC (Agilent 690) equipped with automated liner exchange (ALEX; Gerstel corporation) and cold injection system (CIS; Gerstel corporation) was used for data acquisition. Following data acquisition, raw GC-TOF MS data files were preprocessed and quantification values were reported as peak height.

### Fecal microbiome

Fecal DNA isolation, amplicon sequencing, sequence data analysis and taxonomic classification were performed as we previously described^33^. Briefly, fecal DNA was isolated using the QIAamp DNA stool mini kit (Qiagen, Inc., Germantown, MD) per manufactures instructions. DNA quantity and purity was measured by a Nanodrop spectrophotometer (Nanodrop Technologies, Wilmington, DE) and only samples with DNA concentration greater than 7 ng/μl with OD 260/280 of 1.8–2 were further analyzed for PCR amplification and microbial amplicon sequencing (Novogene, Corp., Sacramento, CA). 16S rRNA V4 region was amplified by PCR using the following primers: 515F (5′-GTGCCAGCMGCCGCGGTAA-3′) and 806R (5′-GGACTACHVGGGTWTCTAAT-3′) and Phusion High-Fidelity PCR Mater Mix (New England Biolabs, Ipswich, MA, USA). Subsequently, the sequencing library was prepared, index codes were added and the library quality was determined. Using Illumina HiSeq2500 platform (Illumina, Inc., San Diego, CA, USA), the library was sequenced and 250 bp paired-end raw reads were generated. Sequence data analysis was performed by mothur (v. 1.39.5)^76^. The trimmed sequences were aligned against the SILVA-based V4 reference alignment, denoised and subjected to chimera removal using UCHIME^77^. Then, the sequences were classified using the Bayesian classifier and Silva non-redundant database v132 as the reference^78^. After exclusion of some sequences (*i.e.* Archaea, chloroplasts, mitochondria, and eukaryotic), the remaining sequences were subjected to OTUs based on at least 97% similarity, which were then classified into taxonomic groups at a threshold of 80%.

### Statistical analysis

Statistical analysis was performed as we previously described^33,66,67,79^. General linear mixed model (IBM SPSS Statistics Version 23, Armonk, NY, USA) was used the analyze the daily and weekly measurements. Diet, time and the interaction of diet and time were included in the model as fixed effects and the pig was considered as random effect with time as repeated measure. As the effect of gender was not significant, therefore it was excluded from the model. Based on the smallest values of fit statistics for corrected Akaike’s Information Criterion and Bayesian Information Criterion, the covariance structure of the repeated measurements for each variable was modeled as either first-order antedependence, autoregressive, heterogenous autoregressive, compound symmetry, heterogenous compound symmetry or toeplitz. The overall growth performance and plasma cytokines were analyzed using univariate GLM procedure (IBM SPSS Statistics Version 23, Armonk, NY, USA). Metabolomics data were analyzed using MetaboAnalyst 3.0^80^ (https://www.metaboanalyst.ca/faces/ModuleView.xhtml) as we previously described^33^. Briefly, data were filtered, the peak height was normalized to the median of all samples and one-way ANOVA test was performed. A PCA was performed to differentiate the metabolites among diets^80^. To determine the effects of dietary groups on the metabolic pathways and metabolite enrichment, a pathway impact analysis was performed. Tukey’s post hoc test was used to separate means between dietary treatments. Differences were considered significant at *P* ≤ 0.05 and a trend at 0.05 < *P* ≤ 0.10.

For microbiota data, the BIOM file generated using Mothur was uploaded to a web-based data visualization tool, MicrobiomeAnalyst (https://www.microbiomeanalyst.ca/)^81^ for downstream statistical analysis and data visualization. NMDS plots were generated at genus level using Bray–Curtis dissimilarity index to assess the overall variation in genus composition (beta diversity). PERMANOVA was used to test the statistical significance of NMDS plots. To assess the richness and evenness of the gut bacterial community in each sample, alpha-diversity analysis was performed. Chao 1 and Observed OTU indices were calculated to measure species richness, while Shannon and Simpson indices were calculated to measure species evenness. To determine the differences in ranking of abundant communities among dietary groups, LDA with LEfSe was performed at the family level using a tool hosted in the Galaxy (server) instance of Huttenhower lab (https://huttenhower.sph.harvard.edu/galaxy/) and the scores were normalized by log10^82^. Bacterial species with an effect size > 2.0 on the logarithmic LDA score was discriminated and considered as populations with distinctly increased numbers, or biomarkers. Kruskal–Wallis and pairwise Wilcoxon tests were performed to measure the effect size of differentially abundant taxon, with differences being considered significant at *P* value ≤ 0.05.

## Supplementary information

Supplementary Information.

## Data Availability

The datasets generated during and/or analyzed during the current study are available from the corresponding author on reasonable request.
