# Supplementary material for: Effect of very low-protein diets supplemented with branched-chain amino acids on energy balance, plasma metabolomics and fecal microbiome of pigs
Source: Sci Rep. 2020 Sep 28;10:15859. doi: 10.1038/s41598-020-72816-8 (PMC7523006; doi:10.1038/s41598-020-72816-8)
Supplement: Supplementary file 1 — Supplementary Information. [file 41598_2020_72816_MOESM1_ESM.docx]

**Effect of Very Low-Protein Diets Supplemented with Branched-Chain Amino Acids on Energy Balance, Plasma Metabolomics and Fecal Microbiome of Pigs**

Shelby Spring^1^, Hasitha Premathilake^1^, Chloe Bradway^1^, Cedrick Shili^1^, Udaya DeSilva^1^, Scott Carter^1^ & Adel Pezeshki^1*^

^1^Department of Animal and Food Sciences, Oklahoma State University, Stillwater, OK 74078, USA

*Correspondence author:

Adel Pezeshki, Ph.D.

Department of Animal and Food Sciences

206C Animal Science Building

Oklahoma State University

Stillwater, OK 74078, USA

Phone: (405) 780-2464

E-mail: adel.pezeshki@okstate.edu

**Supplementary Tables**

|  |
| --- |

**Supplementary Table S1.** Effect of very low protein diets supplemented with branched-chain amino acids on growth measurements

| **Parameters** | **CON^1^** | **LP^1^** | **LP + BCAA^1^** | **SEM^2^** | ***P*-value** |
| --- | --- | --- | --- | --- | --- |
| Initial BW^3^, kg | 8.38 | 8.37 | 8.02 | 0.26 | 0.832 |
| Final BW^3^, kg | 24.56^a^ | 14.54^b^ | 16.93^b^ | 1.07 | < 0.001 |
| ADG^3^, g/d | 599.24^a^ | 228.82^b^ | 329.99^b^ | 36.78 | < 0.001 |
| ADFI^3^, g/d | 1078.10^a^ | 532.31^b^ | 734.01^c^ | 54.88 | < 0.001 |
| G:F^3^, g/g | 0.74^a^ | 0.56^b^ | 0.57^b^ | 0.03 | 0.005 |
| ADPI^3^, g/d | 238.26^a^ | 70.80^b^ | 104.97^b^ | 16.00 | < 0.001 |
| G:P^3^, g/g | 2.54^a^ | 3.23^b^ | 3.10^ab^ | 0.11 | 0.021 |

^1^CON, control diet; LP, low protein diet; LP + BCAA, low protein diet supplemented with branched-chain amino acids. The values are the mean. n=8.

^2^SEM: standard errors of means

^3^BW, body weight; ADG, average daily gain; ADFI, average daily feed intake; G:F, gain:feed ratio; ADPI, average daily protein intake, G:P: gain:protein ratio

^a,b,c^ Within a row, values with different superscripts are different (P *≤* 0.05).

**Supplementary Figures**


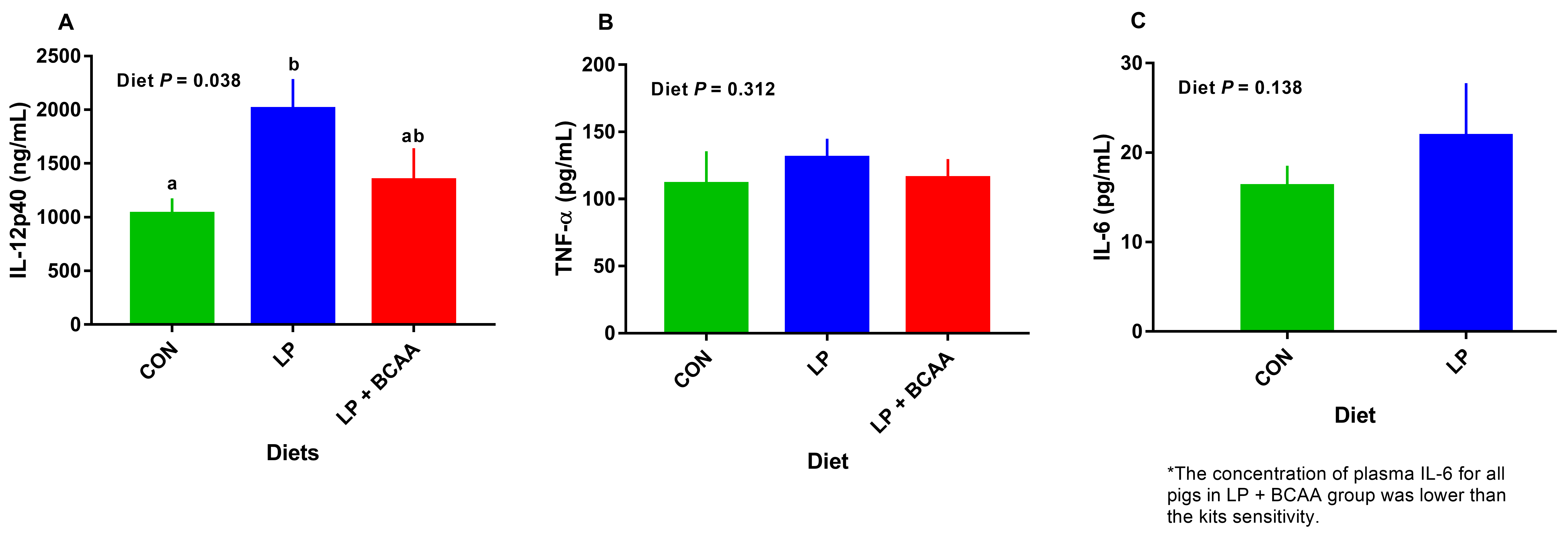


**Supplementary Fig. S1.** **The effect of very low protein diets supplemented with branched-chain amino acids on the concentration of plasma cytokines.**

(**A**) Interleukin 12p40 (IL-12p40), (**B**) Tumor necrosis factor-α (TNF-α), and (**C**) Interleukin-6 (IL-6). For the samples with cytokines concentrations lower than the kit’s sensitivity, values were replaced with the second lowest concentration on the standard curve (the concentration of IL-6 for all plasma samples of pigs in LP + BCAA were lower than the kit’s sensitivity). CON, control diet; LP, low protein diet; LP + BCAA, low protein diet supplemented with branched-chain amino acids. Among groups, values with different superscripts are different (*P* *≤* 0.05). The values are means ± standard errors of means, n=7.

**
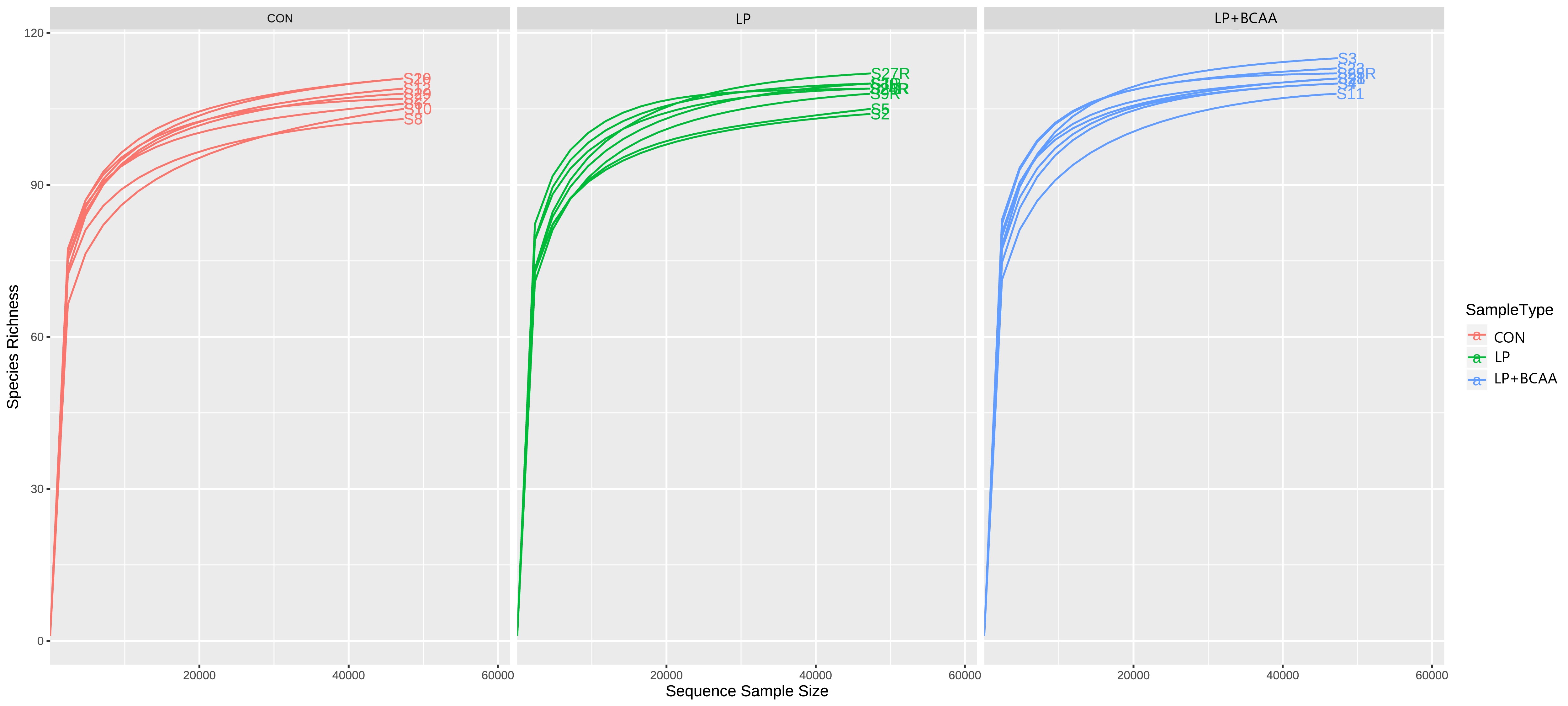
**

**Supplementary Fig. S2. Rarefaction curve analysis for fecal samples collected from pigs fed with very low protein diets supplemented with branched-chain amino acids.**

The rarefaction curve shows the species richness as a function of the number of reads sampled. Each line represents an individual pig. CON, control diet; LP, low protein diet; LP + BCAA, low protein diet supplemented with branched-chain amino acids. n=8.

**B**

**A**

**
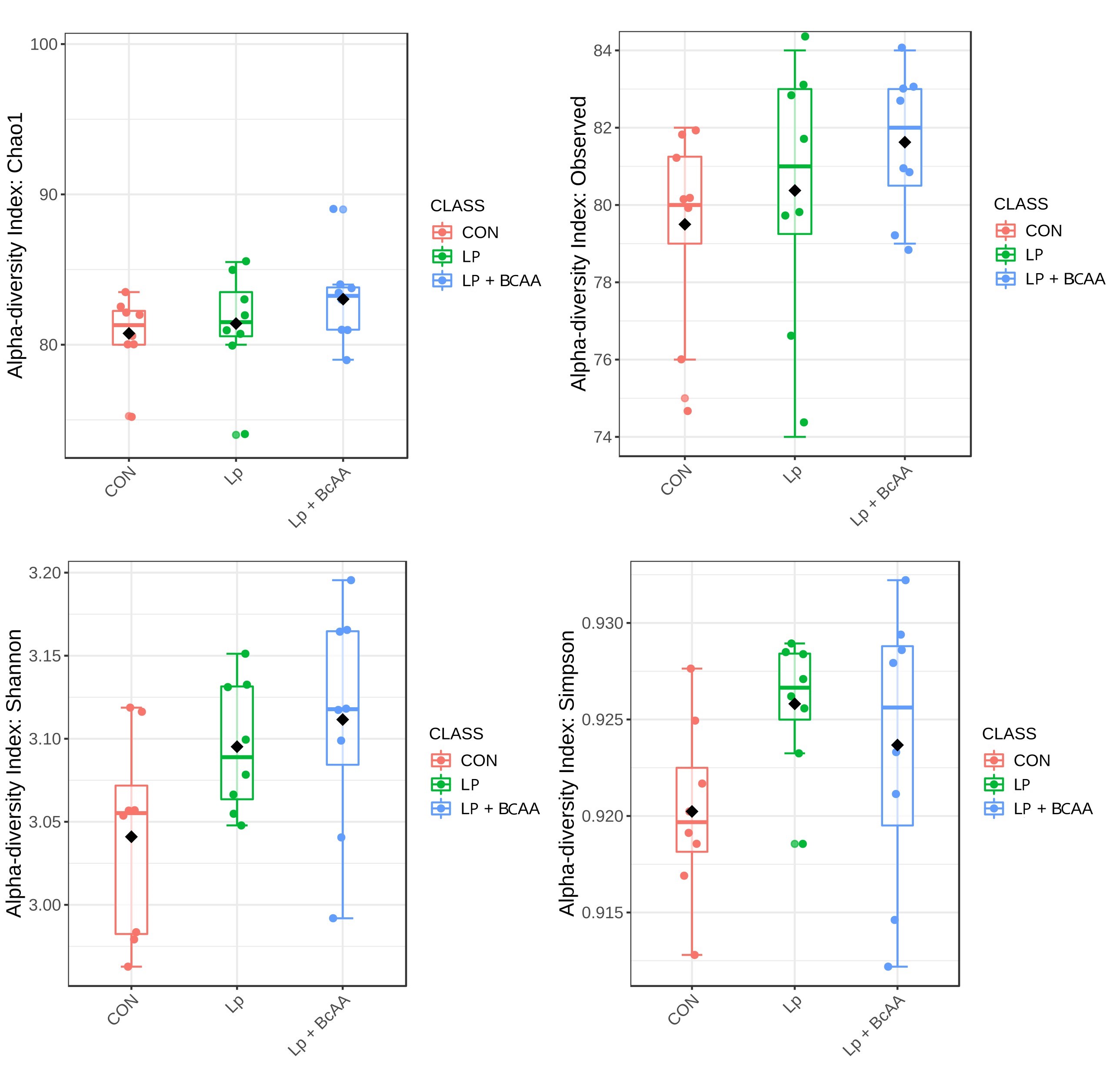
**

**D**

**C**

**Supplementary Fig. S3. Alpha diversity indices for fecal bacterial community in pigs fed with very low protein diets supplemented with branched-chain amino acids.**

1. Chao1, (B) Observed, (C) Shannon, and (D) Simpson. Pigs are grouped based on their dietary treatments, *i.e.* CON, control diet; LP, low protein diet; LP + BCAA, low protein diet supplemented with branched-chain amino acids. Each node represents an individual pig. Differences were considered significant at P *≤* 0.05. The ANOVA P values for Chao1 and Observed indices among dietary groups were 0.638 and 0.153, respectively. The Kruskal-Wallis P values for Shannon and Simpson indices across treatments were 0.075 and 0.120, respectively. n=8.

**A**


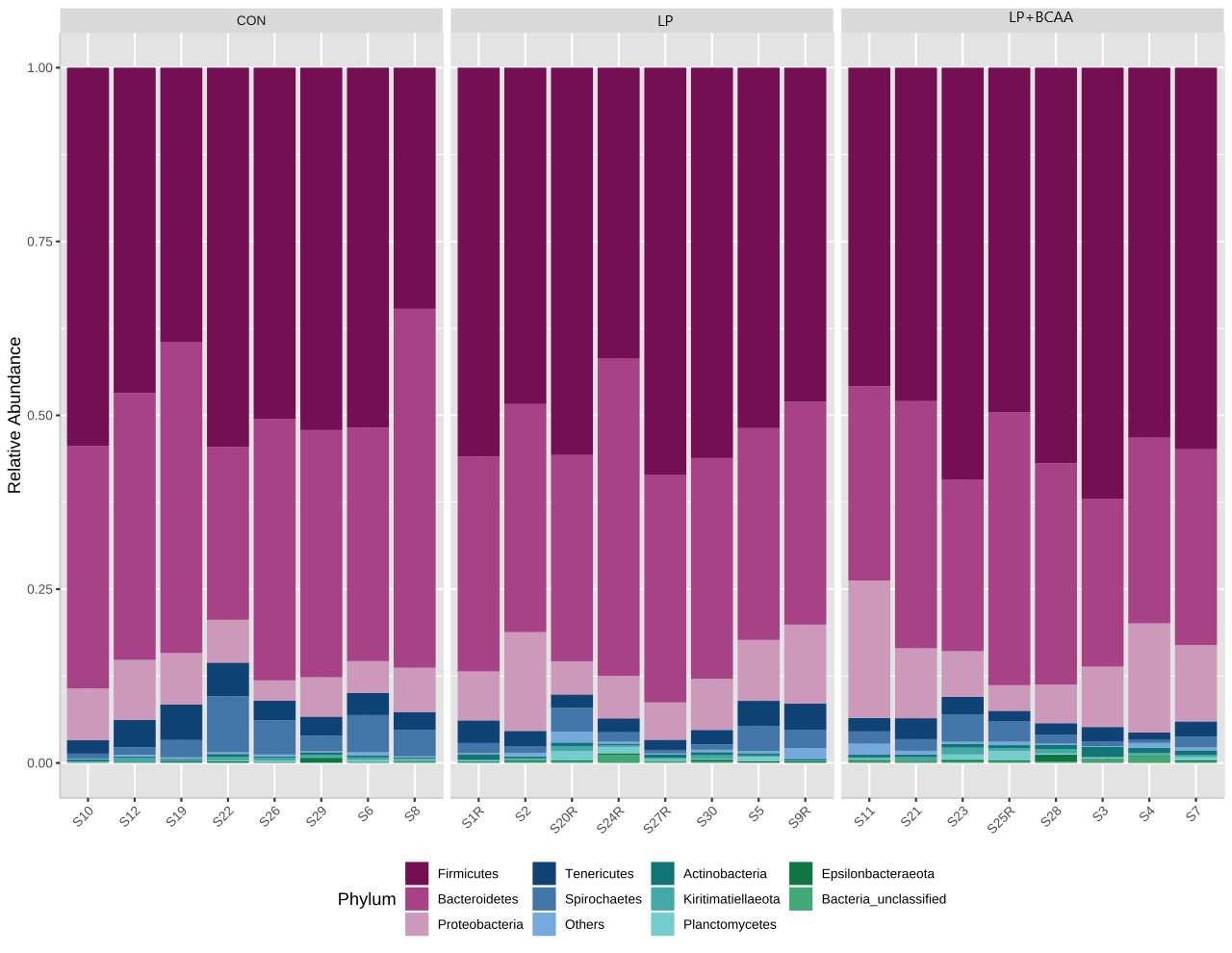


**B**

**
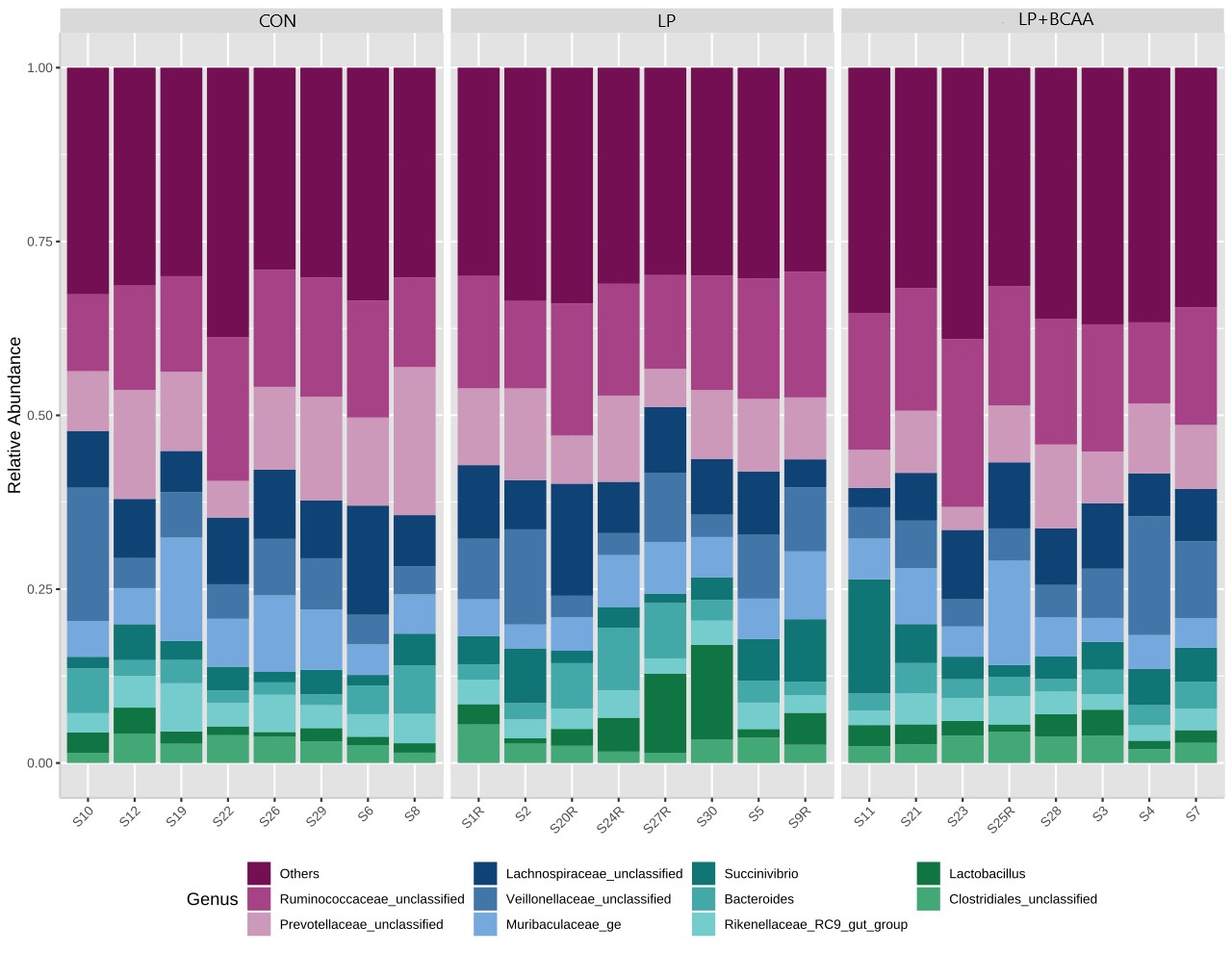
**

**Supplementary Fig. S4.** **The effect of very low protein diets supplemented with branched-chain amino acids on fecal bacterial community of individual pigs at phylum and genus level.**

(**A**) The relative abundance of bacterial community composition at phylum level in fecal samples of individual pigs fed with very low protein diets supplemented with branched-chain amino acids. Only the top 10 phyla are depicted for clarity. (**B**) The relative abundance of bacterial community composition at genus level in fecal samples of individual pigs fed with very low protein diets supplemented with branched-chain amino acids. Only the top 10 genera are depicted for clarity. CON, control diet; LP, low protein diet; LP + BCAA, low protein diet supplemented with branched-chain amino acids. n=8.

**
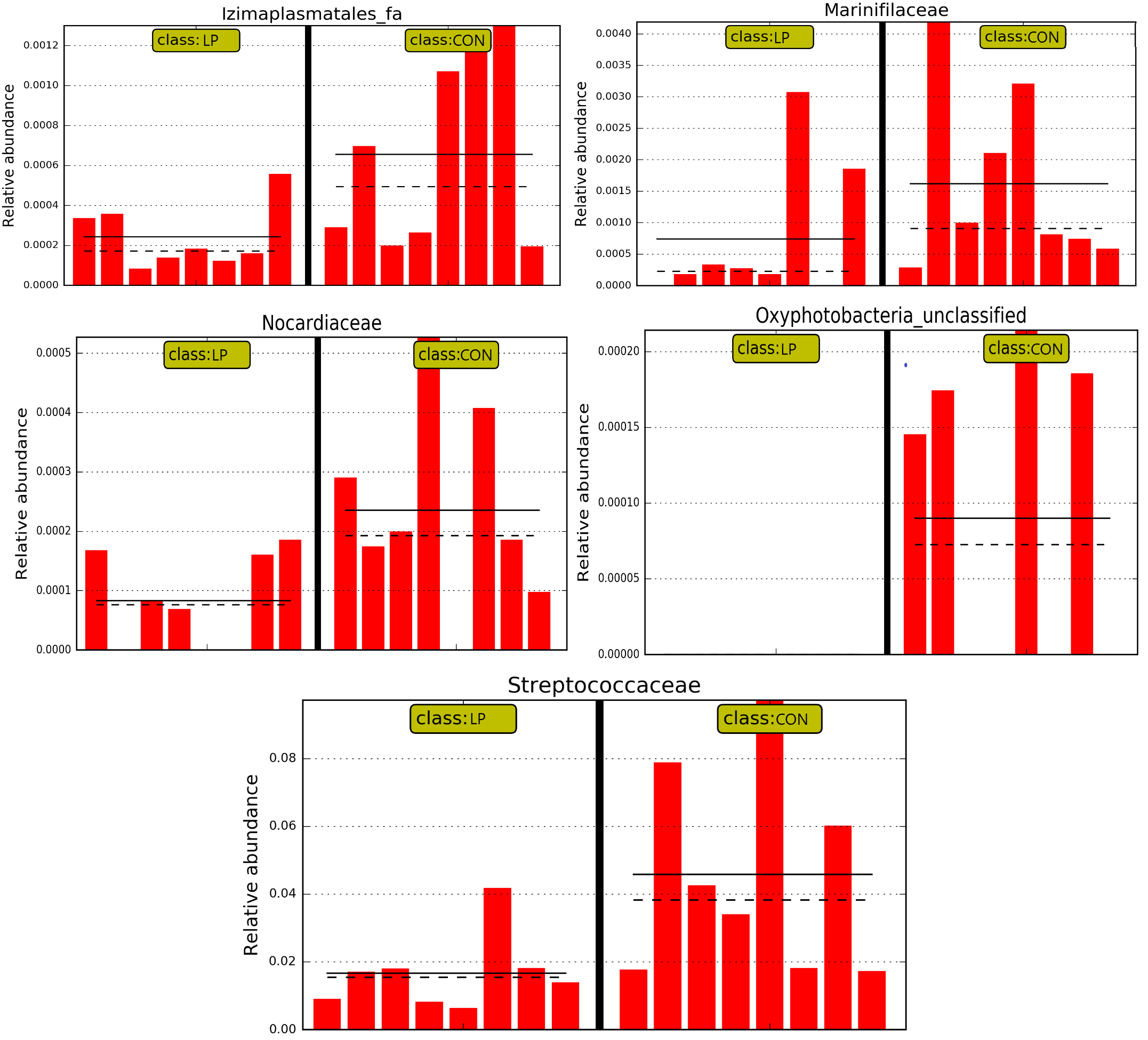
**

**Supplementary Fig. S5. The relative abundance of bacterial communities in feces of pigs fed with control (CON) and low protein (LP) diets using linear discriminant analysis (LDA) with effect size measurements (LEfSe).**

The horizontal lines denote the group means, and the dotted lines mean the group medians. Each bar represents an individual pig. CON, control diet; LP, low protein diet. n=8.

**
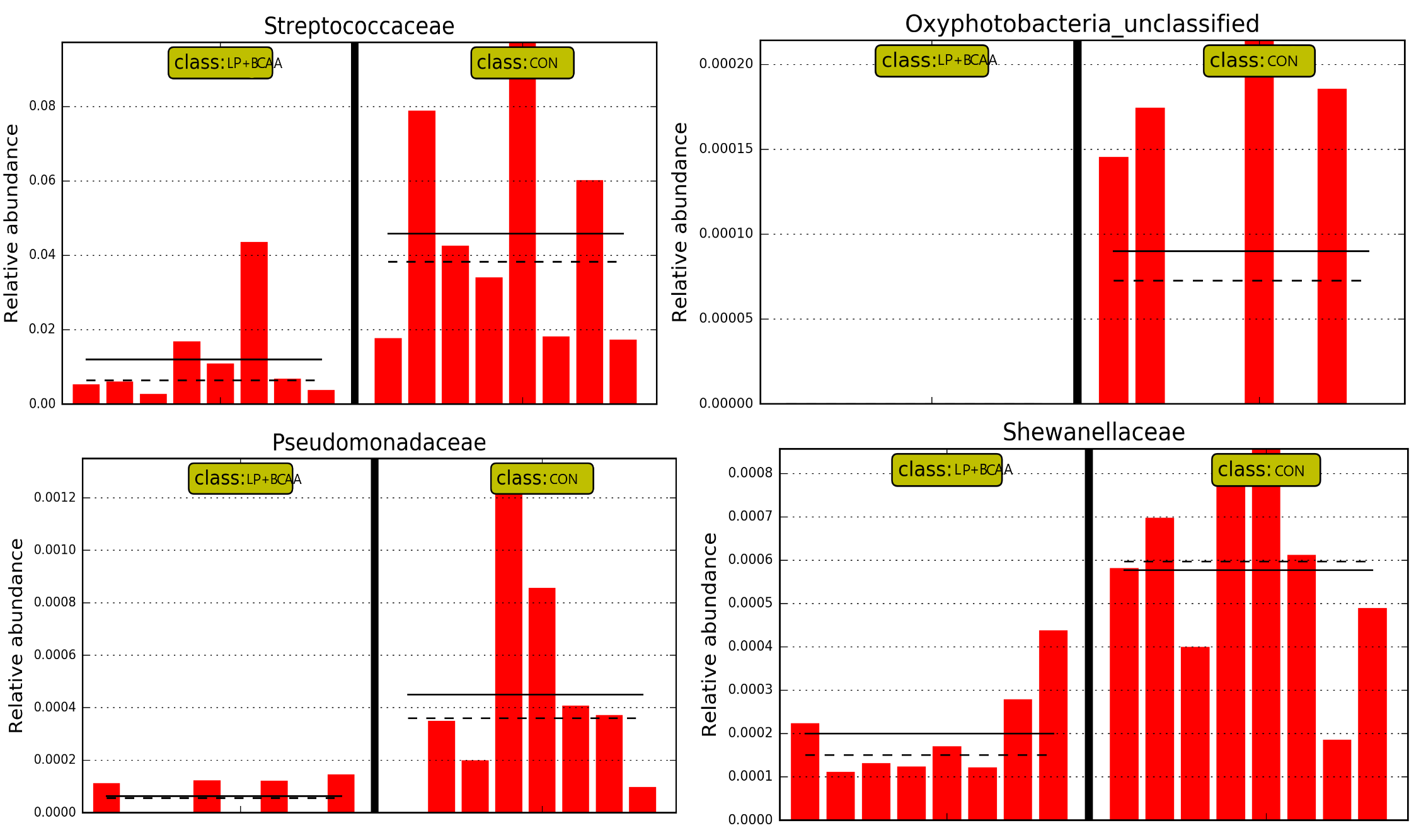
**

**
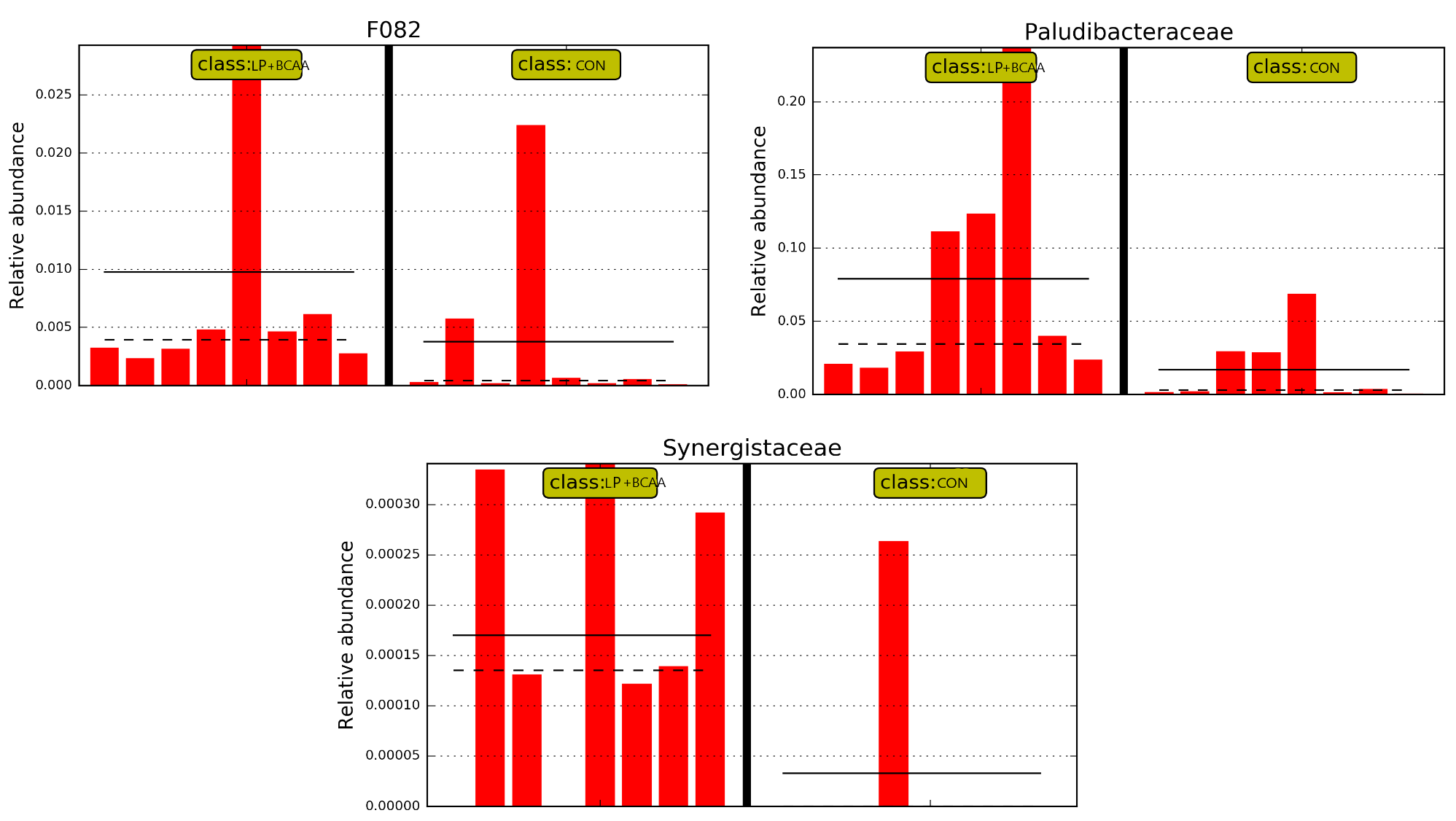
**

**Supplementary Fig. S6. The relative abundance of bacterial communities in feces of pigs fed with control (CON) and low protein diet supplemented with branched-chain amino acids (LP + BCAA) using linear discriminant analysis (LDA) with effect size measurements (LEfSe).**

The horizontal lines denote the group means, and the dotted lines mean the group medians. Each bar represents an individual pig. CON, control diet; LP + BCAA, low protein diet supplemented with branched-chain amino acids. n=8.
